# All That Glitters Is Not Gold: Importance of Rigorous Evaluation of Proteochemometric Models

**DOI:** 10.1021/acs.jcim.5c00395

**Published:** 2025-09-03

**Authors:** Polina Avdiunina, Shamieraah Jamal, Filipp Gusev, Olexandr Isayev

**Affiliations:** † Department of Chemistry, Mellon College of Science, 6612 Carnegie Mellon University, Pittsburgh, Pennsylvania 15213, United States; ‡ Computational Biology Department, School of Computer Science, 6612 Carnegie Mellon University, Pittsburgh, Pennsylvania 15213, United States

## Abstract

Proteochemometric models (PCMs) are used in computational drug discovery to employ both protein and ligand representations jointly for bioactivity prediction. While machine learning (ML) and deep learning (DL) have come to dominate PCMs, often serving as a basis for scoring functions, rigorous evaluation standards have not always been consistently applied. In this study, using kinase-ligand bioactivity prediction as a model system, we highlight the critical roles of data set curation, permutation testing, class imbalances, and various data splitting strategies for mitigating plausible data leakage and embedding quality in determining model performance. Our findings indicate that data splitting and class imbalances are the most critical factors affecting PCM performance, emphasizing the challenges in the generalizing ability of ML/DL-PCMs. We evaluated various protein–ligand descriptors and embeddings, including those augmented with multiple sequence alignment information. However, permutation testing consistently demonstrated that protein embeddings contributed minimally to PCM efficacy. This study advocates for the adoption of stringent evaluation standards to enhance the generalizability of models to out-of-distribution data and improve benchmarking practices.

## Graphical abstract

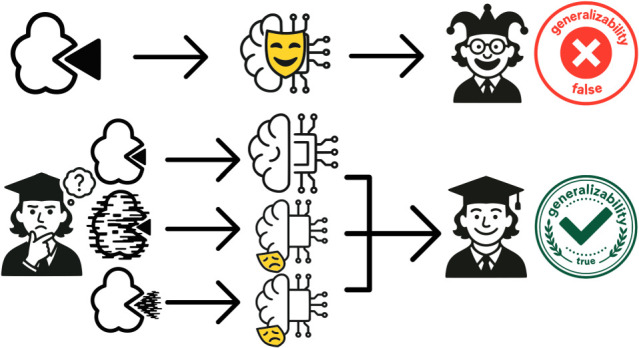

## Introduction

In computer-aided drug design (CADD), virtual screening is a fundamental approach in which large compound libraries are rapidly filtered through multiple iterative rounds to identify hits. Typically, this process involves three main tasks: pose prediction, binding affinity or docking score assessment, and bioactivity prediction, all of which are facilitated by scoring functions (SFs).^1,2^ Traditionally, SFs have employed physics-based, empirical, or knowledge-based methods.^2^ However, the recent rise of machine learning (ML) and deep learning (DL) has shifted the focus toward proteochemometric models (PCMs) that utilize both protein and ligand descriptors to model the ligand-target interaction space.^3–10^ Despite the growing dominance of ML/DL in PCMs, often serving as complex scoring functions, the rigorous evaluation standards essential for their validation are frequently overlooked.

This oversight in rigorous evaluation standards is particularly evident in the selection of commonly used benchmarking data sets. One such benchmark is the Comparative Assessment of Scoring Functions, 2016 (CASF-2016).^11^ It has been demonstrated in numerous studies that several ML/DL models developed using this benchmark exhibit a significant dependency on the inherent similarity between training and test set examples. This results in inflated performance metrics that do not necessarily translate to true predictive power outside the studied data set.^12–14^ The CASF-2016 has been instrumental in revealing these tendencies, but also highlights the need for more robust methods of separating training and validation data sets to prevent model overfitting and ensure genuine generalizability.

Another widely utilized benchmark is the Directory of Useful Decoys Enhanced (DUD-E), which was specifically designed to address the reporting bias where only binding affinities for known binders are typically disclosed, leading to a scarcity of data on nonbinders. The DUD-E data set includes “decoys” for each active ligand at a ratio of 50 to 1, containing over 1.4 million compounds, making it one of the largest databases for benchmarking scoring functions.^15^ This data set, however, has drawn scrutiny due to its method of selecting decoys that are chemically similar yet topologically dissimilar to the active compounds. DUD-E decoys are matched to the physical chemistry of ligands on a target-by-target basis: by the properties of molecular weight, calculated logP, number of rotatable bonds, and hydrogen bond donors and acceptors. Thus, DUD-E decoys were chosen to resemble ligands physically and, therefore, be challenging for docking but at the same time be topologically dissimilar to minimize the likelihood of actual binding. Such bias resulted in models that excel in benchmark tests by exploiting these chemical versus topological discrepancies rather than learning the fundamental principles of protein–ligand interactions. It also led to models with poor generalizability when applied outside the specific context of the data set.^4,5,15,16^ While the use of decoys can be useful, care must be taken in avoiding bias when curating decoy data sets.^6,17^

A second significant source of bias arises from data splitting strategies, which critically influence how models are trained, tested, and their ability to generalize to unseen data. Li and Yang utilized TMAlign^18^ and Needleman-Wunsch^19^ algorithms to cluster proteins within the PDBbind data set based on structural and sequence similarity, respectively. They found that ML-based SFs did not outperform traditional SFs when similar examples were removed from the training data.^20^ Despite these findings, many studies continue to employ random splitting of data sets into training and testing sets, or into cross-validation folds.^8,21,7^ Such approaches do not adequately address the challenge of generalizing models to out-of-distribution (OOD) data (noted by works such as^22^) or to targets not seen during training, underscoring a persistent gap in model evaluation practices.

Moreover, the representation quality of proteins and ligands—essential components of PCMs—often receives insufficient scrutiny.^16^ While various descriptors and embeddings are employed to capture the complex nature of protein–ligand interactions, their effectiveness in truly enhancing model performance remains underexplored. For ligands, common representations include one-hot encoding, SMILES2vec, Morgan Fingerprints, and chemical graphs.^23^ Proteins are often represented by methods such as one-hot encodings, learned embeddings from structure prediction models, molecular graphs, or 3D images, where each channel highlights a different atom type in the binding pocket.^10,24^ Furthermore, protein–ligand interactions might be depicted through voxelized grids or interaction graphs.^21,25,26^ Examples of PCMs utilizing these representations are RFScore,^9^ DeepDTA,^7^ GraphDTA,^27^ AtomNet,^26^ and 3D-KINEssence,^21^ which demonstrate the diverse approaches in modeling but also highlight the variability in their predictive success.

This study investigates the informativeness of protein representations derived from high-quality, structure-guided MSA for proteochemometric models, with a rigorous evaluation of embedding quality and overall model performance. Human kinases serve as the case study since kinase inhibitors represent a major class of FDA-approved therapeutics targeting a wide range of diseases, such as leukemia, breast cancer, nonsmall cell lung cancer, renal cell carcinoma,^28^ as well as various neural and metabolic disorders.^29^ Many ML/DL SFs have also been trained for the purpose of kinase drug discovery,^21,22,7,23^ using the dedicated Davis,^30^ KIBA^31^ or KLIFS^32^ data sets. Therefore, kinases were a natural choice for a deeper investigation of protein representation utility.

We explored various generation and augmentation techniques for protein representations to assess their efficacy in providing informative input for ML/DL models that predict kinase-ligand pair bioactivity. This included rigorous permutation testing of both protein and ligand embeddings to analyze their respective contributions to model performance. Additionally, we explored how class imbalances, data splitting methodologies, and the dimensionality of embeddings influence the performance of these models and assess plausible effects attributed to protein similarities. Our goal is to develop a comprehensive framework for evaluating PCMs that not only exposes and addresses inherent biases but also improves the generalizability and interpretability of these models across diverse data sets.

## Methods

### Data Set

We conducted a comprehensive search for kinase data derived from high-throughput screening panels across various literature sources and open-access databases. The inclusion criteria for data sources were selected to ensure the robustness and relevance of the data for our analyses:

- **Kinase Information**: Due to the frequently encountered inconsistencies in human kinase names and IDs, only human kinases listed in the UniProt^33^ database were included to ensure accuracy and consistency in our data set.
- **Measurement Types**: Data included had to represent one or more of the following experimental measurements: dissociation constant (*K*_d_), inhibition constant (*K*_i_), half-maximal inhibitory concentration (*IC*_50_), *pChEMBL* value, percent inhibition (%*I*), or percent activity (%*A*).
- **Public Availability**: Only data sets with publicly available ligand structures and experimental results were considered, including those providing results as a range of values.

The selected data sources and a comprehensive list of references are provided in the Supporting Information (SI-1.1).

#### Data Curation Protocols

Following the cheminformatic data curation protocol recommended by Fourches et al.,^34^ we manually conducted the initial data cleaning and standardization. Additionally, (i) data marked in the initial sources as associated with fusion proteins and autoinhibitory proteins were excluded to decrease the level of data heterogeneity; (ii) all kinases were carefully identified across all collected sources, with each kinase being assigned a unique identifier, specifically its UniProt ID.

The kinase domain sequences from Modi and Dunbrack’s MSA,^35^ covering 497 human kinase genes, were employed to generate protein descriptors. Only data points for kinases included in the MSA were considered, while the kinases whose sequences were absent from the MSA were excluded from the further analysis. Following a molecular preprocessing workflow: molecule sanitization, normalization, and validation by MolVS v0.1.1; the resulting standardized SMILES strings were then used to generate small molecule descriptors.

After a rigorous curation process, the refined data set was consolidated to 915,998 entries representing 116,936 unique ligands, ensuring a robust foundation for the further machine learning analysis of kinase-ligand interactions.

#### Standardizing Activity Measurements

Experimental activity measurements such as *K*_d_, *K*_i_, and *IC*_50_ can vary numerically by several orders of magnitude. To address this variability and provide a uniform scale for comparison, we introduced a standardization method where the strength of a protein–ligand interaction is characterized by the value of *pX* (*pChEMBL*), calculated using the formula: *pX* = −log _10_(*X*), where *X* represents the numerical value of *IC*_50_, *K*_i_, or *K*_d_ expressed in molar concentration.^36,37^

The evaluation of the biochemical panels’ experimental reproducibility revealed that the average absolute error for identical ligand-protein pairs in *pIC*_50_ data (*pX* for *IC*_50_) is approximately 0.5 units.^38,39^ This observation allows us to indirectly estimate the inherent error in the experimental data, setting a limit on the maximum achievable accuracy of the machine learning models developed from these data (Figure S1).

### Data Featurization

#### Feature Generation for Small Molecules

Small molecule structures were characterized using Circular and Path fingerprints. Circular fingerprints were generated with radii of 3 and 5, encoding the resulting features into binary vectors of 512, 1024, and 2048 bits. Similarly, Path fingerprints were generated with maximum path lengths of 3 and 5, and these features were also encoded into logical vectors of corresponding lengths.

#### Feature Generation for Protein Kinase Domains

The sequences of 497 human kinase domains were extracted from Modi and Dunbrack’s MSA^35^ and were used for the subsequent feature generation. To reduce the noise level in our data set, the TrimAl program^40^ was utilized to selectively filter out columns from the MSA that contained gaps, ensuring a gap-free final alignment. This refined alignment was then used to generate four types of amino acid descriptors: Z-scale,^41^ T-scale,^42^ ST-scale,^43^ and physical properties,^44^ with descriptor vector lengths of 453 for Z-scale, 755 for T-scale, 1208 for ST-scale, and 302 for Physical properties, respectively. Additionally, one-hot encoding of the kinase domain sequences was implemented as a benchmark.

Likewise, we explored sequence and structural embeddings as a way to encode protein target information. Protein embeddings were generated using four different models: ProtBert,^45^ ProtT5,^45^ ESM2,^46^ and AlphaFold2.^47^ The ProtBert, ProtT5 and ESM2 embeddings were generated using Hugging Face library, with embedding dimensions of 1024, 1024, and 1280 per residue, respectively. For structural embeddings, a single representation from AlphaFold2 was extracted during the structure prediction process, which featured an embedding dimension of 384.

#### Padding and Trimming

The sequence/structural embeddings were represented as 2-dimensional matrices ( $R^{L_{\mathrm{seq}}\times c}$ ; *L*_seq_ = length of query sequence, *c* = model embedding dimension). Due to varying lengths of kinase query sequences, embedding sizes were inconsistent across the kinase tree/across kinases. To normalize these lengths and integrate the structural relationships outlined by Modi and Dunbrack,^35^ we implemented a novel padding and trimming procedure (Figure 1). Padding involved adding zero-vectors (1 × *c*) at positions indicated as gaps in the MSA, thereby equalizing the size of all kinase embeddings while creating a uniform “MSA structure” that reflects structural relationships among sequences.

**Figure 1:**
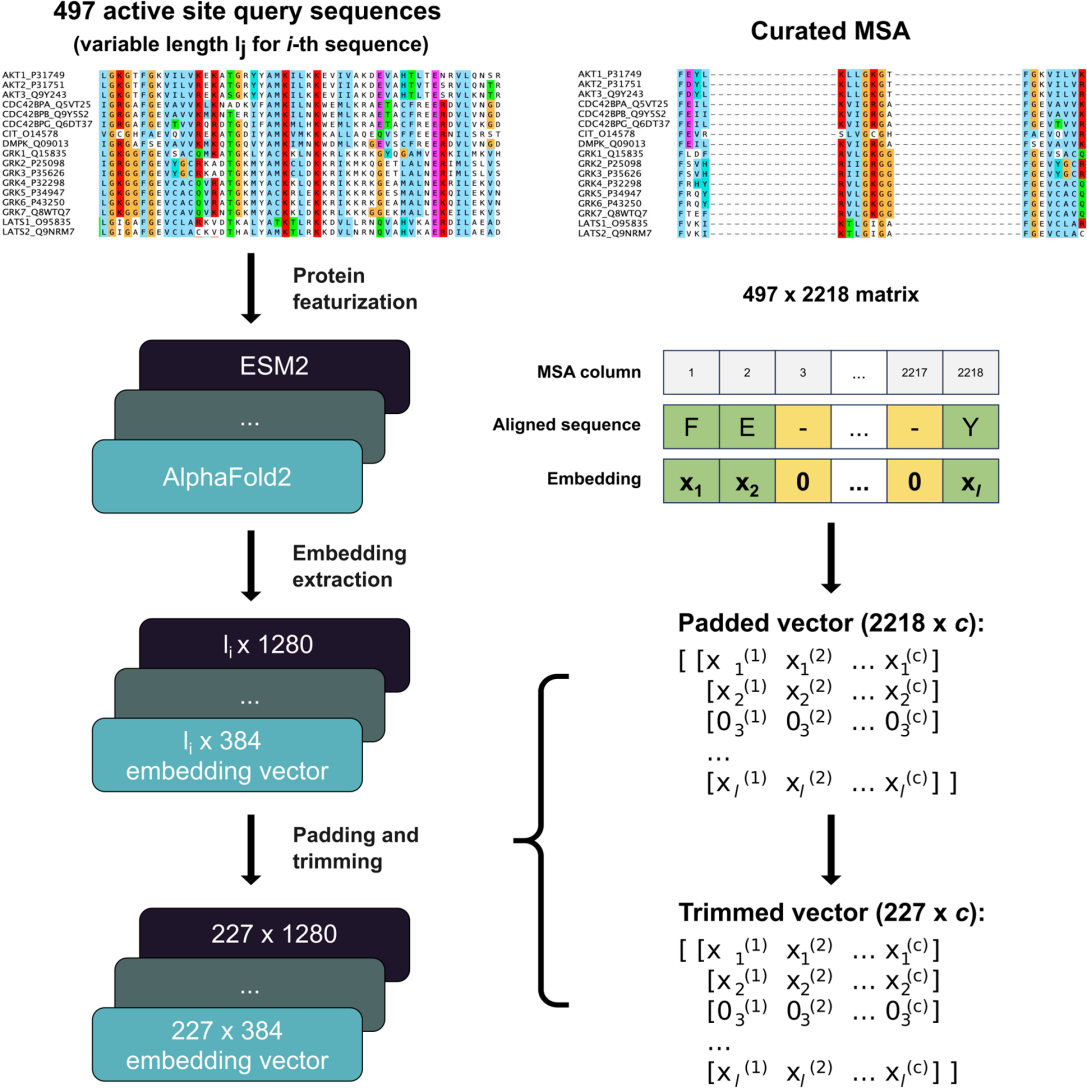
Workflow of protein embedding construction. 497 human kinase domain sequences were used as queries to AlphaFold2, ESM2, ProtT5 and ProtBert models. The final layer embeddings from the models were used as protein representations. Each model produced per-residue embeddings with *c* dimensions (*c* = 384, 1280, 1024, and 1024 respectively). These embeddings were used in ML models described below either as is or after padding and trimming.

Trimming was conducted using TrimAl^40^ and aimed to reduce matrix sparsity by selectively removing MSA columns where a high percentage of sequences contained gaps. We established various trimming thresholds (10, 15, 20, 85, 90, and 95%), ultimately setting a 95% threshold for all subsequent analyses to optimally balance data richness with matrix density (Figure S4).

### Baseline Models

For baseline models, we performed data sampling and selected two data sets, which were used for the subsequent modeling. The first data set combines data from the kinase families ABL, CDK, and ALK, which were identified as the most data-rich within our study. The second data set included data on 10 human kinases that were best represented in terms of experimental data within the final curated data set.

Feature vectors for small molecules were generated using Circular and Path molecular fingerprints (radii of 3 and 5, and path lengths of 3 and 5 respectively). These fingerprints were encoded into vectors of varying lengths (512, 1024, and 2048 bits) to capture different levels of structural information.

For protein representation, feature vectors were derived using four types of amino acid descriptors (Z-scale, T-scale, ST-scale, and Physical properties), as well as one-hot encoding of kinase sequences and ProtBert protein embeddings.

These feature vectors were then combined to form a comprehensive set of kinase domain-ligand pair descriptors. In total, we generated 144 unique combinations of kinase domain-ligand representations based on the described methodologies.

For building the baseline models, we utilized the scikit-learn package^48^ and constructed 144 proteochemometric models employing both Random Forest and XGBoost techniques with 100 trees each, employing default settings for maximum tree depth (*max_depth*) and split quality measurement functions (*criterion*). Stratified 5-fold cross-validation was applied to ensure reliable model validation and to assess the generalizability of each model. The performance of these models was evaluated through stratified 5-fold cross-validation, focusing on *R* ^2^ and mean absolute error (MAE) as our primary metrics.

### Learnable Protein Representation Models

#### Dimensionality Reduction

To maintain the integrity of kinases and kinase family relationships while managing the dimensionality of AlphaFold2 and ESM2 embeddings, we trained a deep convolutional autoencoder (CAE). The CAE treated each embedding as a single-channel array, allowing convolutional blocks to capture specific patterns within the embeddings. The architecture featured an encoder with multiple convolutional layers — each followed by batch-normalization and max-pooling to reduce and stabilize the input dimensions. After passing through the convolutional layers, the output was flattened and then passed through a dense layer to obtain a latent embedding. A decoder then reconstructed the input from the latent embedding using a symmetrical setup to the encoder. The training loss combined mean-squared error (for embedding reconstruction accuracy) and cross-entropy (for kinase family classification accuracy), ensuring the latent space accurately represented the clustering of kinase families while retaining reconstructive details. Specific implementation details are provided in Table S3.

#### Model Building and Evaluation

For proof-of-concept and to streamline our model-building efforts, we selectively subsetted the data set to focus on wild-type kinase data from the three largest families: CMGC, TYR, and TKL. To establish a classification framework, the total number of 24,491 points were binarized with a threshold of *pX* = 6 (Figure 2). Ligands were featurized with Morgan fingerprints with the radius and the number of bits set to 5 and 1024 respectively. Protein embeddings were concatenated with ligand fingerprints to create joined protein–ligand representations.

**Figure 2:**
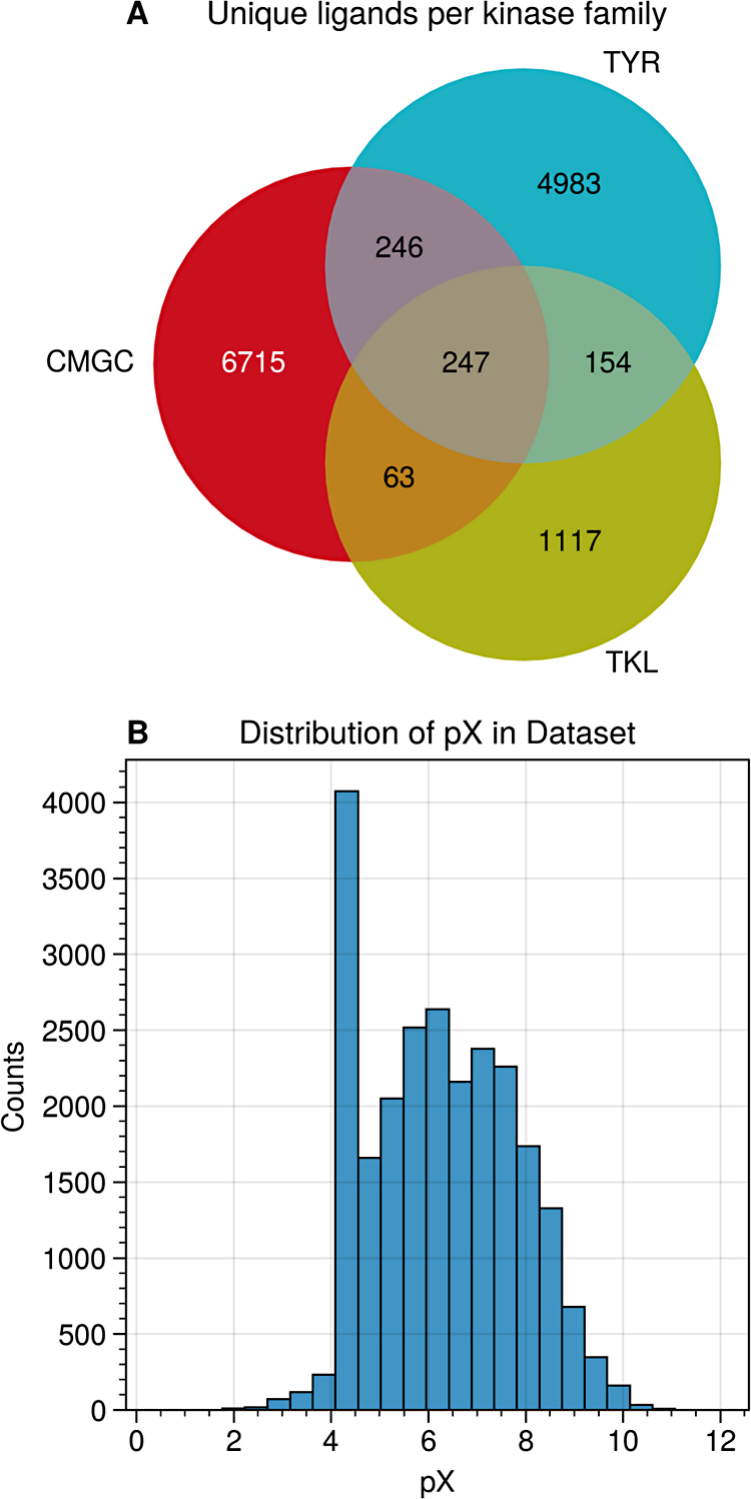
Overview of the data set used in the study. (A) Distribution of data points and unique ligands across different kinase families, highlighting the data density and diversity. (B) Histogram of pX values with the distribution of values across different bins.

To test model robustness we employed three data splitting strategies: a random split for maximum data variance exposure, a kinase-based split to eliminate kinase leakage, and a family based split to prevent family bias in training data. Models were built using XGBoost, optimized with hyperparameter tuning via Optuna^49^ (See SI for details). For each experimental condition, the optimal set of parameters was identified and used to train the best model using internal cross-validation splits appropriate to the chosen data splitting strategy. Multilayer perceptron (MLP) classifiers were also trained in a similar manner, but showed no performance improvement against XGBoost (averaged across original embeddings F1 of 0.77 vs 0.85) and were more computationally demanding, so we proceeded with all analyses using only XGBoost models.

The final trained models were evaluated with the F1 score, receiver operating characteristic (ROC), area under ROC (AUROC) and Recall metrics on the holdout set. The feature importances were calculated to determine the relative contributions of protein and ligand features within the models. Protein feature importance (PFI) was defined as a fraction of total feature importance related to the protein part of the joined representation.

Additionally, permutation tests were conducted to assess the individual influence of protein and ligand embeddings, as well as labels on model performance. This involved shuffling protein embeddings in the training set and evaluating model performance against unshuffled embeddings in the holdout set. This method was mirrored by shuffling embeddings in the test sets to understand the direct effects of permutations on model predictive ability.

#### Stochastic Negative Addition

Following the methodology outlined by Brocidiacono et al.,^6^ our models incorporated Stochastic Negative Addition (SNA) approach to test the impact of data balancing on model performance. Initially, to gather a sufficient pool of inactive ligands we sampled the ChEMBL database, excluding any ligands already present in our data set.

After establishing our cross-validation splits, each training and validation fold was individually rebalanced by calculating the current ratio of inactive to active ligands for each kinase, determining the number of additional inactive ligands needed to achieve a 1:1 ratio, and, if necessary, randomly selecting the appropriate number of ligands from the ChEMBL pool. These selected ligands were then labeled as ‘inactive’ and added to the data set, aiming to rigorously test the models’ ability to generalize and perform under more balanced class distributions, thereby potentially reducing bias and enhancing the reliability of bioactivity predictions.

#### Statistical Analysis

Our models were evaluated through 5-fold cross-validation for models trained under random and kinase-based splits, and 3-fold cross-validation for those under the family based split. In each cross-validation cycle, performance metrics were computed on the holdout fold, and results were aggregated across cycles using the mean and the standard deviation to compare model performance. To determine the statistical significance of the results, the statsmodels package was used to run ANOVA for multiple variables (including the following performance metrics: F1, ROCAUC, Recall, PFI) and Tukey’s HSD tests for posthoc pairwise comparisons with adjusted p-values. A result was considered statistically significant if the p-value was less than 0.05. In all other cases, all variables (data split, embedding, trimming, SNA, permutations and permutation phase) were taken into account as stated.

## Results

### Baseline Models

#### ABL, CDK, and ALK Kinase Families

Among the 144 models constructed, the top 10 with the lowest mean absolute error (MAE) were selected for detailed analysis, with results presented in Figures 3A,B. The *R* ^2^ values for these models varied from 0.77 to 0.82, with no consistent patterns indicating a preference for specific parameters or descriptors that significantly outperformed others.

**Figure 3:**
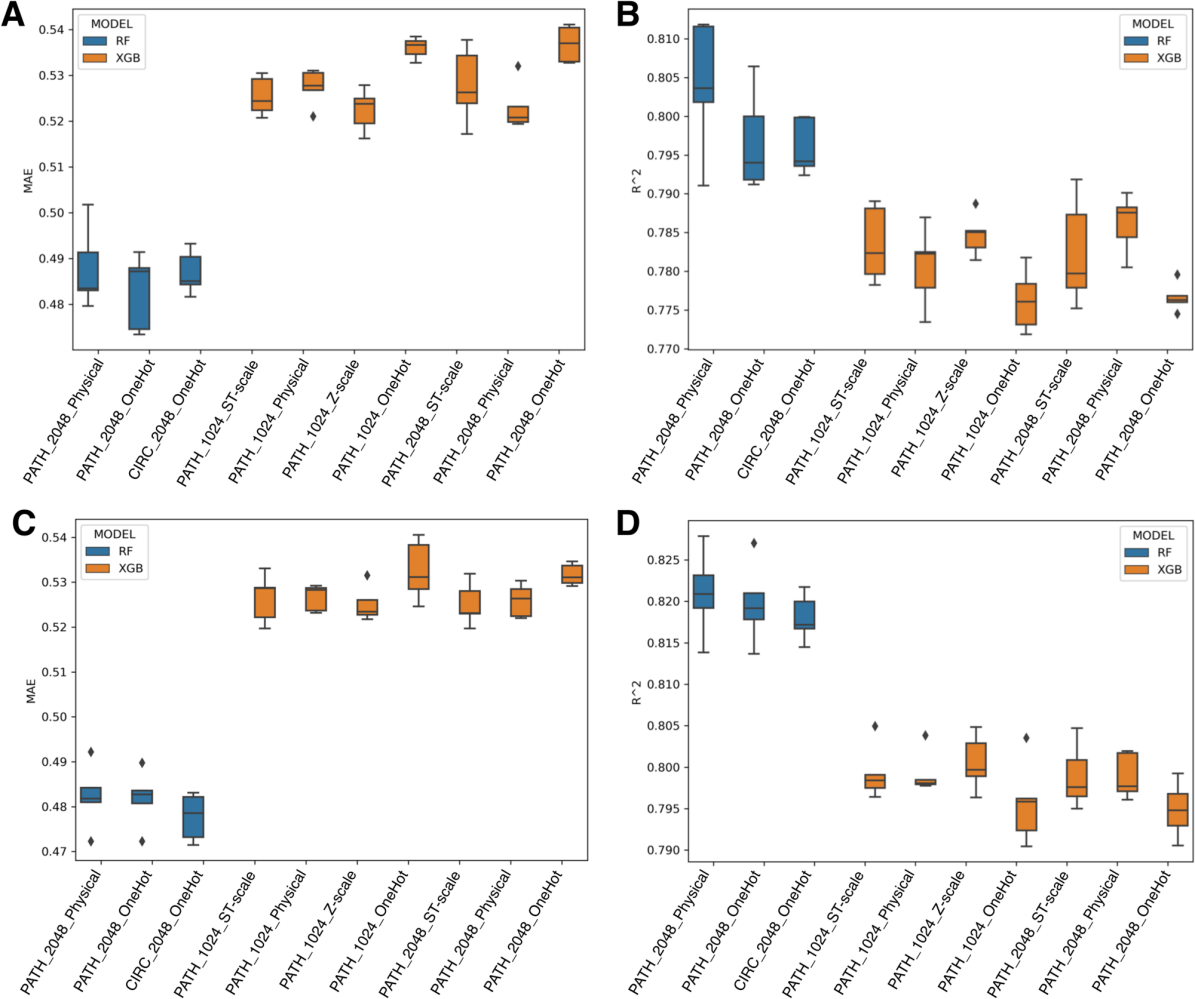
Distribution of MAE and *R* ^2^ for baseline models. The *X*-axis categorizes various parameter combinations and descriptor types used in constructing the models, including Circular fingerprint (CIRC) and Path fingerprint (PATH) paired with vector lengths (512, 1024, and 2048 bits) and different amino acid descriptors (Physical, Z-scale, T-scale, ST-scale, or one-hot encoding). Panels (A) and (B) show the MAE and *R* ^2^ distribution, respectively, for the best ten models of ABL, CDK, and ALK kinase families. Panels (C) and (D) display the MAE and *R* ^2^ distribution for the top 10 represented protein kinases’ best ten models.

It was observed that the *R* ^2^ values for models built with the Random Forest algorithm were consistently higher than those obtained using the XGBoost algorithm, albeit within a narrow margin of up to 0.03. Similarly, the MAE values were generally lower for Random Forest models, suggesting superior performance over XGBoost in this data set.

#### Top 10 Represented Human Protein Kinases

We hypothesized that the minimal impact of protein primary sequence information on model performance might be attributed to kinase domain sequences remaining highly conservative within each family. To test this hypothesis, we conducted another round of modeling using the data from the top 10 most representative human kinases that were selected based on their coverage in the final data set. The full list of selected kinases is provided in the SI. The methodology for data preparation was similar to the one used for the ABL, ALK, and CDK kinase families. Using Random Forest and XGBoost methods, we developed 144 proteochemometric models, with the top 10 models according to MAE identified and detailed in Figures 3C,D.

Despite high *R* ^2^ values ranging from 0.79 to 0.83, no definitive patterns emerged from the analysis that could distinctly highlight a successful set of parameters and descriptors. The *R* ^2^ values for models built with Random Forest were consistently higher than those using the XGBoost algorithm, with variations within a narrow range (up to 0.03). Moreover, the MAE values for XGBoost were greater than those for Random Forest, suggesting a superior performance of Random Forest in these evaluations.

Notably, for both baseline model groups, embedding vectors for protein sequences were not among the top performers, indicating a lower information content compared to protein descriptors. The occasional inclusion of one-hot encoded sequence models in the top ranks further underscores that protein embeddings contribute little additional predictive value.

Figure S2 presents the full distributions of MAE and *R* ^2^ across all 144 models for both classes of baseline models, offering further insight into the variability and comparative performance of Random Forest and XGBoost.

### Analysis of Embeddings

Due to the inherent sequential and structural similarities among kinases, capturing their distinctive features for targeted ligand design presents a desirable yet challenging task. Modi and Dunbrack^35^ facilitated this process by curating a multiple sequence alignment guided by kinase secondary structure regions, potentially enabling the capture of subtle variations in domain architectures. This refined MSA served as a valuable prior for adapting protein embeddings generated from AlphaFold2, ESM2, ProtT5 and ProtBert models. Following padding and trimming procedures, these embeddings were flattened, scaled, and subsequently visualized using Uniform Manifold Approximation and Projection (UMAP). As depicted in Figure 4, the analysis reveals a significant impact of padding, which effectively streamed evolutionary information from MSA to protein embeddings and aligned clusters with kinase family annotations. Trimming, while slightly dispersing these clusters, maintained the overall structural configuration. For a more detailed discussion of the structure of the protein embedding latent space, the readers are directed to the Supporting Information.

**Figure 4:**
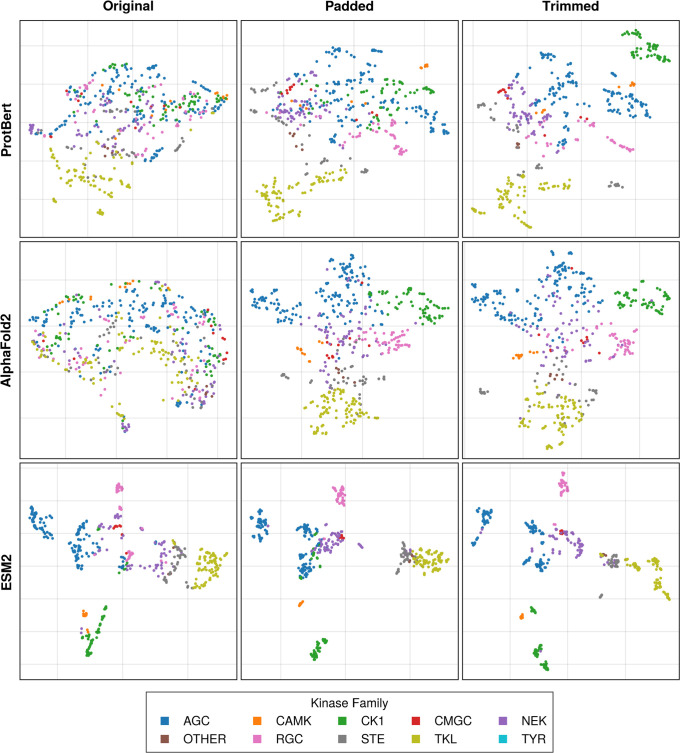
UMAP projections of protein embeddings across the kinase tree. The transformation of protein embeddings from ProtBert, AlphaFold2, and ESM2 models through padding and trimming procedures is shown from the left to the right. The original embeddings (left column) were padded (middle column) by inserting zero-vectors at residue positions indicated as gaps in the MSA. The embeddings were then trimmed (right column) to minimize sparsity, by removing positions that contained gaps in over 95% of sequences. Each model’s embeddings are visualized on a UMAP, color-coded by kinase family, to highlight the clustering effects of these modifications.

### Dimensionality Reduction

The initial embeddings generated by the AlphaFold2, ProtBERT, ProtT5 and ESM2 models were of impractically large dimensions for efficient ML model training, with flattened outputs of 87168, 232448, 232448, and 290560 dimensions, respectively. This vast dimensionality raised the risks of overfitting, given more features than data points.

Typically, this challenge is addressed through mean-aggregation, where averages are calculated across the embedding dimensions, resulting in more manageable vector sizes of 384, 1024, 1024, and 1280 for AlphaFold2, ProtBert, ProtT5 and ESM2 respectively. This aggregation was applied to both the original and the trimmed embeddings, with the results shown in Figure 5. As anticipated, mean-aggregation led to a significant loss of positional context for each amino acid, obscuring critical structural information.

**Figure 5:**
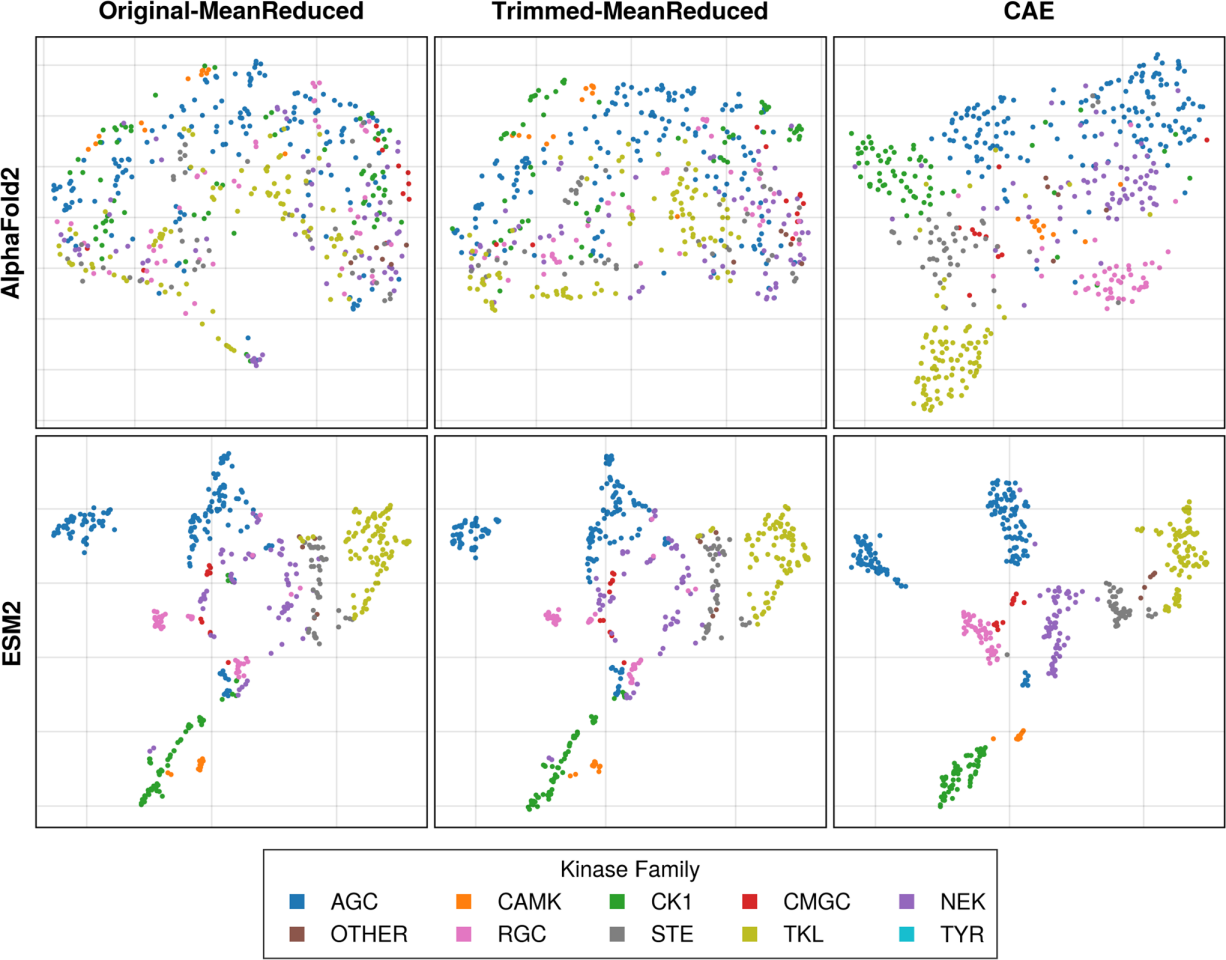
UMAP projections of assessed dimensionality reduction techniques. This figure illustrates the effects of mean-reduction on AlphaFold2 and ESM2 embeddings before and after applying a convolutional autoencoder (CAE). Initially, both original and trimmed embeddings underwent mean-reduction to facilitate model training, obscuring critical structural relationships between kinases (left two panels).To mitigate this information loss, a CAE was employed to reduce dimensions while ensuring that kinases’ relationships in the latent space were preserved.

Indeed, we find a loss of distinctive clustering in AlphaFold2 embeddings when mean-reduction is performed (Figure 5). The ESM2 embeddings, however, preserve local kinase relationships in all cases. Additionally, we used a convolutional autoencoder (CAE) to process each embedding into a low-dimensional latent space to capture the relationships between kinases. This process yielded latent vectors sized 192 for AlphaFold2 and 1680 for ESM2, effectively preserving family wise relationships within the latent space, as demonstrated in Figure 5.

### Impact of Embedding Modifications on PCM Model Performance

Our initial analysis showed no significant differences among the AlphaFold2, ProtBert, ProtT5, and ESM2 embeddings under baseline conditions—where neither class rebalancing (SNA) nor permutation was applied—across all data splits, embedding modifications, and performance metrics. As shown in Table 1, embedding type did not significantly affect F1 score based on a four-way ANOVA. Although AUROC differences were statistically significant, post hoc Tukey’s HSD tests revealed no meaningful pairwise differences that would indicate a clear preference for any embedding type (Table S5). Similarly, Recall did not vary across embeddings in pairwise comparisons (Table S6), except in scenarios with SNA applied, where ProtT5 outperformed AlphaFold2 by a small margin (0.02).

**Table 1:** Statistical Analysis of XGBoost Models after Cross-Validation across Metrics and with or without Rebalancing the Dataset^a^,^b^

	baseline ANOVA			ANOVA without rebalancing			ANOVA with rebalancing		
metric	variable	*F*	*p*-value	variable	*F*	*p*-value	variable	*F*	*p*-value
F1 score	data split	363.59	***†	data split	106.87	***†	data split	186.99	***†
	embedding	0.08	n.s.†	embedding	3.10	*	embedding	5.07	** .
	trimming	0.43	n.s.†	trimming	0.18	n.s.†	trimming	7.28	**
	SNA	0.24	n.s.†	permutation	1120.67	***†	permutation	3144.14	***†
				permutation phase	108.90	***†	permutation phase	131.88	***†
AUROC	data split	661.41	***†	data split	1033.51	***†	data split	236.55	***†
	embedding	2.00	n.s.†	embedding	4.52	**	embedding	0.49	n.s.†
	trimming	1.24	n.s.†	trimming	0.95	n.s.†	trimming	0.18	n.s.†
	SNA	186.35	***†	permutation	1582.19	***†	permutation	2097.63	***†
				permutation phase	247.08	***†	permutation phase	18.01	***†
recall	data split	1294.37	***†	data split	1346.88	***†	data split	68.96	***†
	embedding	8.43	***	embedding	23.86	***	embedding	8.05	***†
	trimming	7.03	***	trimming	3.28	n.s.†	trimming	3.79	*
	SNA	2686.70	***†	permutation	81.02	***†	permutation	54.47	***†
				permutation phase	832.82	***†	permutation phase	549.99	***†
protein feature importance	data split	217.25	***†	data split	300.70	***†	data split	561.91	***†
	embedding	191.75	***†	embedding	125.47	***†	embedding	1593.74	***†
	trimming	45.40	***†	trimming	57.66	***†	trimming	112.19	***†
	SNA	2.40	n.s.†	permutation	136.63	***†	permutation	340.83	***†
				permutation phase	1.00	n.s.†	permutation Phase	16.69	***

a Baseline ANOVA refers to the analysis performed without taking into account permutations of embeddings. ANOVA was also performed with all permutation conditions with and without rebalancing.

b Significant p-values are indicated with asterisks. *p < 0.05, **p < 0.01, ***p < 0.001. Significant conditions were followed up with posthoc Tukey’s HSD tests for pairwise comparisons (Tables S4–S7). Values with † indicate that posthoc comparisons validated the significance (or lack thereof) of a variable.

The observed differences in PFI were primarily attributable to embedding size rather than embedding type. Since feature importances were summed across embedding dimensions, larger embeddings (e.g., ESM2) yielded higher PFIs than smaller ones (e.g., AlphaFold2), independent of underlying model architecture. This pattern was consistent across data splits and conditions (Table S10), suggesting that model performance was minimally affected by the specific embedding model and was instead influenced by the dimensional scale of the input features.

We also evaluated whether incorporating structural insights from Modi and Dunbrack’s MSA^35^—via embedding padding and trimming—could enhance model performance. However, neither approach produced significant improvements in F1 score, AUROC, or Recall when comparing the original and modified embeddings (Tables 1 and S4–S6). While trimming did significantly affect PFI across different SNA conditions and data splits, pairwise comparisons revealed only a minor average improvement of 0.02, observed specifically in the kinase and family splits (Tables S7 and S10). These results suggest that although padding and trimming adjust the structural representation of protein embeddings, they do not substantially enhance model predictive performance.

### Impact of Data Splitting Strategies on PCM Model Performance

The data splitting strategy had the most profound effect on the model performance metrics (Table 2). Posthoc pairwise comparisons revealed that models trained using a random split consistently outperformed those using kinase-specific or family-specific splits in terms of F1 score, as shown in Figure 6, Recall and AUROC, as illustrated in Figures S10 and S5. A similar behavior (Figures S8 and S18) was observed for the Matthews correlation coefficient (MCC), a preferable metric for imbalance data, but frequently underutilized in practice.^50^ This also held true with or without SNA (Figures S7 and S5), except for the F1 score (Figure 6) and PFI (Figure S6) which were not significantly different between kinase and family splits in the baseline (no rebalancing and no permutation) conditions. After rebalancing, however, the same trend was observed as the overall result (discussed further below). This confirmed that the structural relationship among protein targets across the training and testing folds contributes to model performance and undermines generalizability to unseen targets.

**Table 2:** Statistical Analysis of XGBoost Models after Cross-Validation across Metrics and Data Splits Using ANOVA^a^

**metric**	**data split**	**variable**	*F*	***p*-value**
F1 score	random	embedding	0.34	n.s.†
		trimming	7.37	***
		SNA	622.50	***†
	kinase	embedding	0.41	n.s.†
		trimming	0.44	n.s.†
		SNA	50.04	***†
	family	Embedding	0.12	n.s.†
		trimming	0.09	n.s.†
		SNA	13.50	***†
AUROC	random	embedding	2.62	*
		trimming	14.33	***
		SNA	303.45	***†
	kinase	embedding	2.36	n.s.†
		trimming	4.27	*
		SNA	351.02	***†
	family	embedding	0.80	n.s.†
		trimming	0.00	n.s.†
		SNA	21.57	***†
recall	random	embedding	1.13	n.s.†
		trimming	3.79	*
		SNA	2368.98	***†
	kinase	Embedding	0.94	n.s.†
		trimming	1.51	n.s.†
		SNA	1119.46	***†
	family	embedding	11.05	***
		trimming	6.06	**
		SNA	1360.79	***†
PFI	random	embedding	543.53	***†
		trimming	4.78	*
		SNA	59.12	***
	kinase	embedding	103.83	***†
		trimming	39.67	***†
		SNA	25.21	***
	family	embedding	10.39	***†
		trimming	12.33	***†
		SNA	7.03	***†

a Significant p-values are indicated with asterisks and were followed up with posthoc Tukey’s HSD tests for pairwise comparisons (Tables S8–S11). *p < 0.05, **p < 0.01, ***p < 0.001. Values with † indicate that posthoc comparisons validated the significance (or lack thereof) of a variable.

**Figure 6:**
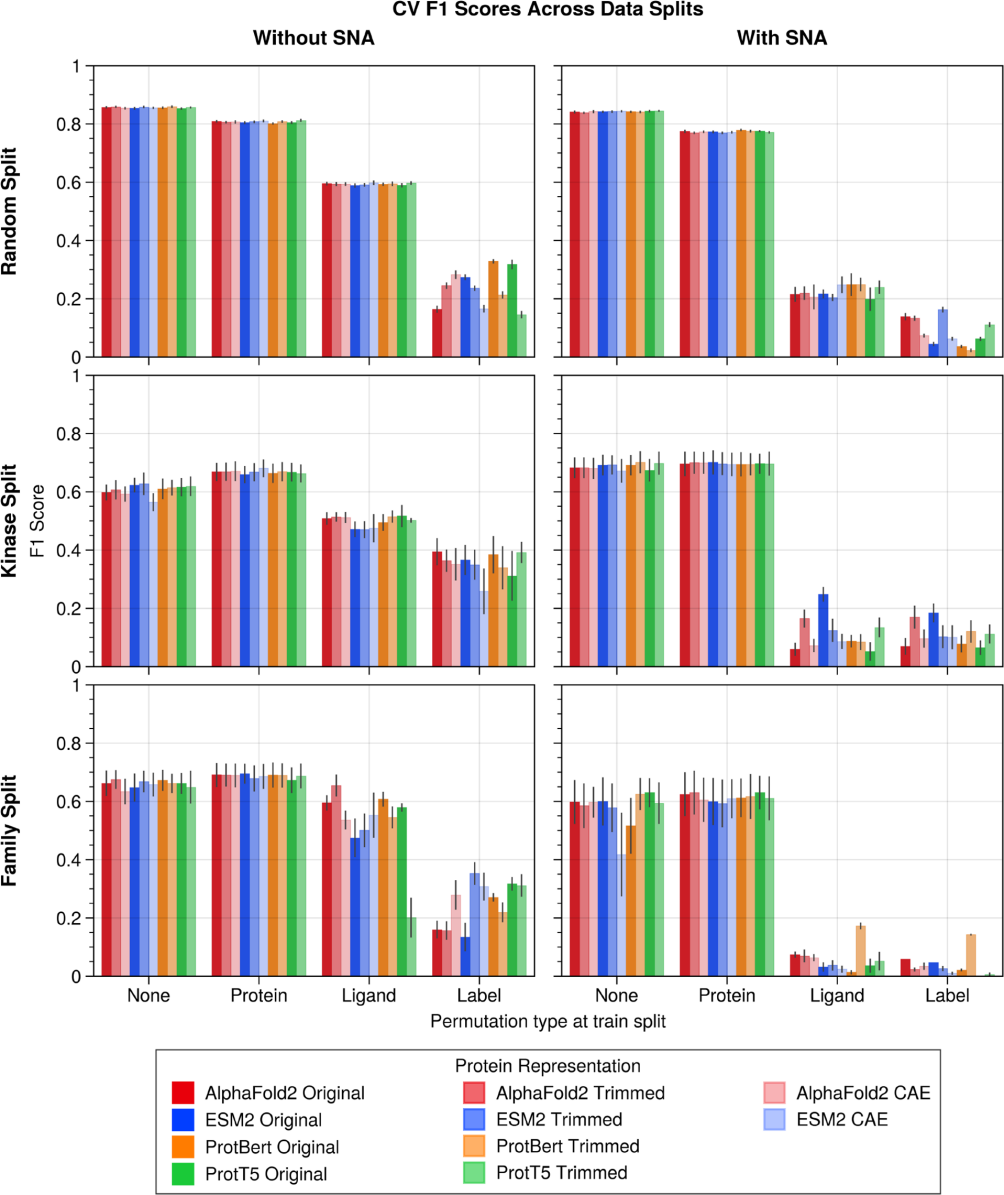
Performance of XGBoost models trained to predict bioactivity of kinase-ligand pairs as measured by F1 Score (mean ± s.e.) across cross-validation folds. Different cross-validation splits were created using either random split (top row), kinase split (middle row) or family split (bottom row) strategies. Models were trained with the data set as is (left column) or after rebalancing with SNA (right column) using a ratio of 1:1 inactives to actives. AUROC (Figure S5), PFI (Figure S6), Recall (Figure S7) and MCC (Figure S8) are shown in the corresponding supplementary figures. Embeddings or labels were also permuted during training (depicted on *X*-axis) and compared with the baseline (no permutation; “None”). Test set permutations were reported separately: F1 (Figure S9), AUROC (Figure S10), PFI (Figure S11), Recall (Figure S12) and MCC (Figure S13).

### Effect of Dimensionality Reduction on PCM Model Performance

To address potential information loss due to mean-aggregation of the original and trimmed embeddings, we explored whether dimensionality-reduced versions, specifically AlphaFold2 CAE and ESM2 CAE, could affect the model performance. Our analysis, however, indicated no significant performance change between the dimensionally reduced embeddings and their original or trimmed counterparts across various data splitting strategies, as detailed in Tables S4–S10. Notably, in some cases, the number of dimensions significantly impacted PFI, with AlphaFold2 CAE demonstrating a lower PFI compared to AlphaFold2 original and trimmed embeddings, and ESM2 CAE showing a higher PFI relative to ESM2 original and trimmed versions. However, no consistent dependency was observed across different scenarios (Tables S7 and S10). Moreover, there were no significant differences in PFI between ESM2 CAE and its original or trimmed versions across kinase and family splits, suggesting that the impact of protein features on model performance diminishes when protein similarities between the train and test splits are minimized. This reinforces the earlier observation that reducing impact of close evolutionary relationships among targeted proteins improves model generalizability and mitigates the influence of excessive embedding dimensions on model output.

### Effect of Permutation Testing

To assess whether our model predictions truly arise from learning protein and ligand features, we conducted systematic permutation tests. These included shuffling protein and ligand embeddings, as well as activity labels, during either training or testing, to evaluate their impact on model performance.

Protein permutation had little effect on F1 score and AUROC (Figures 6 and S5), suggesting the model may not strongly rely on protein features. However, a noticeable drop in MCC (Figures S8 and S13) and PFI (Figure S6) suggests that protein information is still contributing meaningfully.

Ligand permutation had a much larger impact, significantly lowering F1, AUROC, and MCC values—especially driving MCC scores to the range of random prediction—highlighting the importance of ligand embeddings, likely due to their greater chemical diversity.

Label permutation during training produced some surprising results: recall actually increased in certain conditions, suggesting that the model, when learning from random labels, overpredicts positives (Table S6). Notably, recall was more affected by protein shuffling without SNA, but by ligand shuffling with SNA, indicating different reliance patterns depending on class imbalance.

Shuffling during testing generally had less impact than shuffling during training. However, protein shuffling at test time caused a stronger drop in F1 and AUROC (Figures S9 and S10), confirming the protein’s role in generalization. AUROC consistently declined across all splits when labels were permuted—regardless of when the shuffling occurred—suggesting this metric remains more robust to noise than others.

### Impact of Stochastic Negative Addition on PCM Model Performance

Brocidiacono et al.^6^ recently proposed Stochastic Negative Addition (SNA) as a method to improve generalization by randomly sampling decoy compounds unlikely to bind a target. Our implementation of SNA followed a similar strategy, randomly drawing ChEMBL compounds that were not previously seen, assuming low binding probability.

SNA significantly affected AUROC, Recall, and PFI, with minimal effect on F1 (Table 1). Across all data splits, SNA improved AUROC by 0.09 and Recall by 0.32 (Tables S5 and S6), while F1 score and PFI remained mostly unchanged (Tables S4 and S7), consistent with earlier findings that AUROC may inflate performance under class imbalance without improving actual classification. At a per-split level (Table 2), F1 score remained stable overall but varied slightly: decreasing in the random split (−0.01), increasing in the kinase split (+0.07), and dropping in the family split (−0.08). In contrast, AUROC rose most sharply in kinase and family splits (+0.18), and Recall showed similar gains (kinase: + 0.48, family: + 0.45), with only a minor increase in the random split (+0.045).

These results imply that SNA is particularly beneficial under stricter generalization scenarios (i.e., kinase/family splits), where the model’s ability to correctly identify positives improves significantly—without artificially boosting F1 or PFI. This supports SNA as a valuable strategy for improving generalization, though it does not appear to increase reliance on either protein or ligand features per se.

## Discussion

A critical examination of current PCM practices reveals several key concerns. First, models evaluated using random splits often overestimate generalizability,^14^ performing substantially better than those evaluated with protein family based splits. This discrepancy suggests that many published models may be overly optimistic in their reported performance, especially for out-of-distribution targets. Second, permutation testing consistently shows that protein embeddings contribute minimally to model performance compared to ligand features, calling into question the added value of complex protein representations. Third, improvements in metrics like AUROC through techniques such as Stochastic Negative Addition often fail to translate to meaningful improvements in more balanced metrics like F1 score or MCC, suggesting that some reported advances may be artifacts of evaluation methodology rather than genuine improvements.

Our results confirm these trends. Models trained on random splits outperformed those using kinase or family splits, though performance within kinase families remained feasible, despite the integration of MSA-based kinase relationships. Previously, Li et al.^51^ developed a ‘leak-proof’ version of the PDBbind data set, explicitly excluding proteins and ligands with high similarity to the training set from the test set. This approach led to the retraining of several established scoring functions, including AutoDock Vina,^52^ RFScore,^53^ DeepDTA^7^ and InteractionGraphNet,^54^ which then performed substantially better than initially reported.

In our study, we focused on the construction of embeddings and the strategic use of permutation testing to evaluate model robustness and performance. The literature offers a variety of protein and ligand representations for ML and DL-based scoring functions. While “Y-permutation” is widely documented, our research highlights the advantages of “X-permutation”, demonstrating the minimal impact of protein permutations on model outcomes as opposed to the significant disruptions caused by ligand and label permutations. Similarly, Gorantla et al.^55^ assessed the impact of random permutations of ligand and protein representations on binding affinity predictions with neural network models using Davis^30^ and KIBA^31^ data sets. Models trained with randomly permuted protein contact maps had performed on par with those trained on conventional 1D or 2D embeddings across various metrics. Conversely, when ligand representations were permuted, a marked decrease in performance was observed, in line with our observations.

This provides additional evidence that it is the ligand information that is pivotal for bioactivity prediction using PCM methods. The possible reason for this is the greater diversity found in ligand embeddings compared to protein embeddings, enabling models to extract more useful patterns from the distribution of ligand data. This observation aligns with the well-established utility of ligand-based QSAR models. Consistent with other studies,^5,12,56,16^ this underscores the significance of ligand features and reveals how data set bias and potential overfitting predominantly influence model performance, rather than protein–ligand interactions. Therefore, we encourage researchers developing PCM models and deep learning-based scoring functions to implement ablations, permutation tests, and thorough statistical analyses to validate their findings.

In an attempt to improve the information content of our protein embeddings, we incorporated kinase phylogeny through a detailed structural analysis conducted by Modi and Dunbrack.^35^ While this served to discern both intra- and interfamily relationships among kinases, our models did not show improved generalization to unseen kinase families. Nonetheless, generalization within kinase families was successful to some extent, suggesting that models might perform better within more closely related groups, however, broader kinome-wide predictions remain challenging.

This also highlighted the general issue that embedding methods like AlphaFold2, ProtT5, ProtBert, and ESM2 generate high-dimensional outputs that are hard to integrate into PCM workflows. Processing them through feed-forward or convolutional layers before prediction may also uncover data set biases rather than true meaningful relationships between the inputs and outputs.^52^ Although our use of a convolutional autoencoder helped reduce dimensions and capture essential relational data, the resulting embeddings still lacked necessary information to improve model performance.

One promising direction is to enrich protein embeddings using strategies that explicitly account for conformational diversity. Recent work^57^ has explored generating protein conformational ensembles through MSA clustering in AlphaFold2, as well as training models to predict docked complex structures, such as in AlphaFold3 ^58^ or DynamicBind.^59^ Protein embeddings that incorporate such conformation-specific information may gain variability and richness comparable to ligand embeddings, potentially enhancing their predictive value in PCM models. A latent space that captures the subtle differences/nuances between proteins could also significantly enhance the modeling of bioactivity. Lin et al.^46^ demonstrated this concept by developing an encoder-quantization-decoder model that constructs an amino acid vocabulary (ProTokens), enabling rapid structural comparisons consistent with physical constraints and pairwise alignments.

Finally, the results from employing Stochastic Negative Addition also highlighted unexpected outcomes. While the AUROC and Recall scores confirm SNA’s utility, especially in more curated data splits, detailed examination of the F1 score indicated no substantial improvement in model performance. Notably, MCC is often more informative than other metrics in imbalanced contexts because it will be low if the model only learns to predict the majority class. Our study reinforces that MCC is a preferable metric for imbalanced data that is unfortunately underutilized in practice. Additionally, protein feature importances remained static post-SNA, indicating that data augmentation did not boost the utility of protein embeddings. Balancing the data set before model construction is crucial nevertheless, as evidenced by its beneficial impact on ligand-based QSAR and c-RASAR models, preventing model overfitting and promoting more reliable predictions.^44,60^

For the field to progress meaningfully, researchers must abide by stricter standards in model development and evaluation. This entails careful data curation protocols, realistic data-splitting techniques reflecting intended use cases, thorough ablation studies and permutation tests to evaluate feature contributions, and a variety of complementary performance metrics. Perhaps most critically, researchers should approach seemingly impressive results with healthy skepticism, especially those arising from evaluation protocols that may not accurately represent the challenges of real-world applications.

Ultimately, PCM modeling should focus not on achieving high benchmark scores on data sets with known biases, but rather on serving as effective screening tools that can reliably predict activities for new protein–ligand pairs in real-world drug discovery settings. By adhering to the methodological suggestions while remaining aware of statistical artifacts, researchers can create models that genuinely leverage the complementary information present in protein and ligand data, thereby improving computational drug design.

## Supplementary Material





## Data Availability

Our data and source code are available at https://github.com/isayevlab/pcm_glitter

## Notes

Tables S4–S11, raw data: statistical analysis results, including ANOVA (ZIP)
SI Methods: details on data set curation, the baseline model data set, hyperparameter tuning, and the implementation of the CAE; Table S1, model performance on the ABL, CDK, and ALK kinase families across all 144 kinase–compound prediction models; Table S2, model performance on the top 10 kinases across all 144 kinase–compound prediction models; Table S3, architecture of CAE used for dimensionality reduction; Figures S1–S13, additional performance evaluations of machine learning models, and UMAP projections of ESM2 and AlphaFold2 structural embeddings under different trimming thresholds (PDF)
