# Supplementary material for: All That Glitters Is Not Gold: Importance of Rigorous Evaluation of Proteochemometric Models
Source: J Chem Inf Model. 2025 Sep 3;65(19):10239–52. doi: 10.1021/acs.jcim.5c00395 (PMC12529762; doi:10.1021/acs.jcim.5c00395)
Supplement: Supplementary file 2 [file ci5c00395_si_002.pdf]

Supporting Information: All that glitters is not gold: Importance of rigorous evaluation of proteochemometric models

Polina Avdiunina,[†,¶] Shamieraah Jamal,[‡,†,¶] Filipp Gusev,[‡,†] and Olexandr Isayev[*,†,‡]

†*Department of Chemistry, Mellon College of Science, Carnegie Mellon University, Pittsburgh, PA, 15213*

‡*Computational Biology Department, School of Computer Science, Carnegie Mellon University, Pittsburgh, PA, 15213*

¶*These authors contributed equally to this work.*

E-mail: olexandr@olexandrisayev.com

Contents

SI-1 Methods **S-4**

SI-1.1 Dataset curation S-4

SI-1.2 Baseline model dataset S-4

SI-1.3 Analysis of embeddings S-20

SI-1.4 Hyperparameter tuning S-21

SI-1.5 Implementation of Convolutional Autoencoder S-23

References **S-34**

List of Figures

S1 5-fold cross-validation parity plot for Random Forest for ABL kinase family . S-5
S2 Violin plots of model performance across all 144 kinase–compound prediction models S-6
S3 Pairwise analysis of kinase domain structures S-22
S4 UMAP projections of ESM2 and AlphaFold2 structural embeddings with varied trimming thresholds S-24
S5 Performance of XGBoost models by AUROC S-25
S6 Performance of XGBoost models by PFI S-26
S7 Performance of XGBoost models by Recall S-27
S8 Performance of XGBoost models by MCC, permutations on the train set . . S-28
S9 Performance of XGBoost models by F1 score, permutations on the test set . S-29
S10 Performance of XGBoost models by AUROC, permutations on the test set . S-30
S11 Performance of XGBoost models by PFI, permutations on the test set S-31
S12 Performance of XGBoost models by Recall, permutations on the test set . . S-32
S13 Performance of XGBoost models by MCC, permutations on the test set . . . S-33

List of Tables

S1 Model performance on the ABL, CDK, and ALK kinase families across all 144 kinase–compound prediction models S-7
S2 Model performance on the top 10 kinases across all 144 kinase–compound prediction models S-14
S3 The architecture of CAEs used to reduce the dimensions of 2D embeddings . S-23

SI-1 Methods

SI-1.1 Dataset curation

Data for this study were carefully extracted, standardized, and processed from seven different sources, primarily focused on the aggregation and storage of medicinal-chemical information. The data distribution analysis across these sources revealed that 59.2% of the entries originated from four major online databases: BindingDB contributed 26.6%[S1], DiscoverX Project accounted for 20.9%, the ChEMBL database provided 8.9%[S2], and the NIH LINCS Library comprised 2.9%[S3]. The remaining 40.58% of the dataset entries were derived from two key publications: Christmann-Franck et al. accounted for 98.4% of these[S4], while Duong-Ly et al. contributed 1.6%[S5].

SI-1.2 Baseline model dataset

For the ABL, CDK and ALK kinase families dataset, the selected kinases include ABL1 (Tyrosine-protein kinase ABL1), BTK (Tyrosine-protein kinase BTK), ITK (Tyrosine-protein kinase ITK), ABL2 (Tyrosine-protein kinase ABL2), BMX (Cytoplasmic tyrosine-protein kinase BMX), CSK (Tyrosine-protein kinase CSK), TEC (Tyrosine-protein kinase Tec), FES (Tyrosine -protein kinase Fes), FER (Tyrosineprotein kinase Fer), TXK (Tyrosine-protein kinase TXK), MATK (Megakaryocyteassociated tyrosine-protein kinase), CDK2 (Cyclin-dependent kinase 2), CDK4 (Cyclin-dependent kinase 4), CDK9 (Cyclin-dependent kinase 9), CDK5 (Cyclin-dependent-like kinase 5), CDK6 (Cyclin-dependent kinase 6), CDK1 (Cyclin-dependent kinase 1), CDK7 (Cyclin-dependent kinase 7), CDK16 (Cyclin-dependent kinase 16), CDK17 (Cyclin-dependent kinase 17), CDK11B (Cyclin-dependent kinase 11B), CDK14 (Cyclin-dependent kinase 14), CDK3 (Cyclin-dependent kinase 3), CDK18 (Cyclin-dependent kinase 18), CDK8 (Cyclin-dependent kinase 8), CDK19 (Cyclin-dependent kinase 19), CDK15 (Cyclin-dependent kinase 15), CDK13 (Cyclin-dependent kinase 13), CDK10 (Cyclin-dependent kinase 10), CDK12 (Cyclinde-

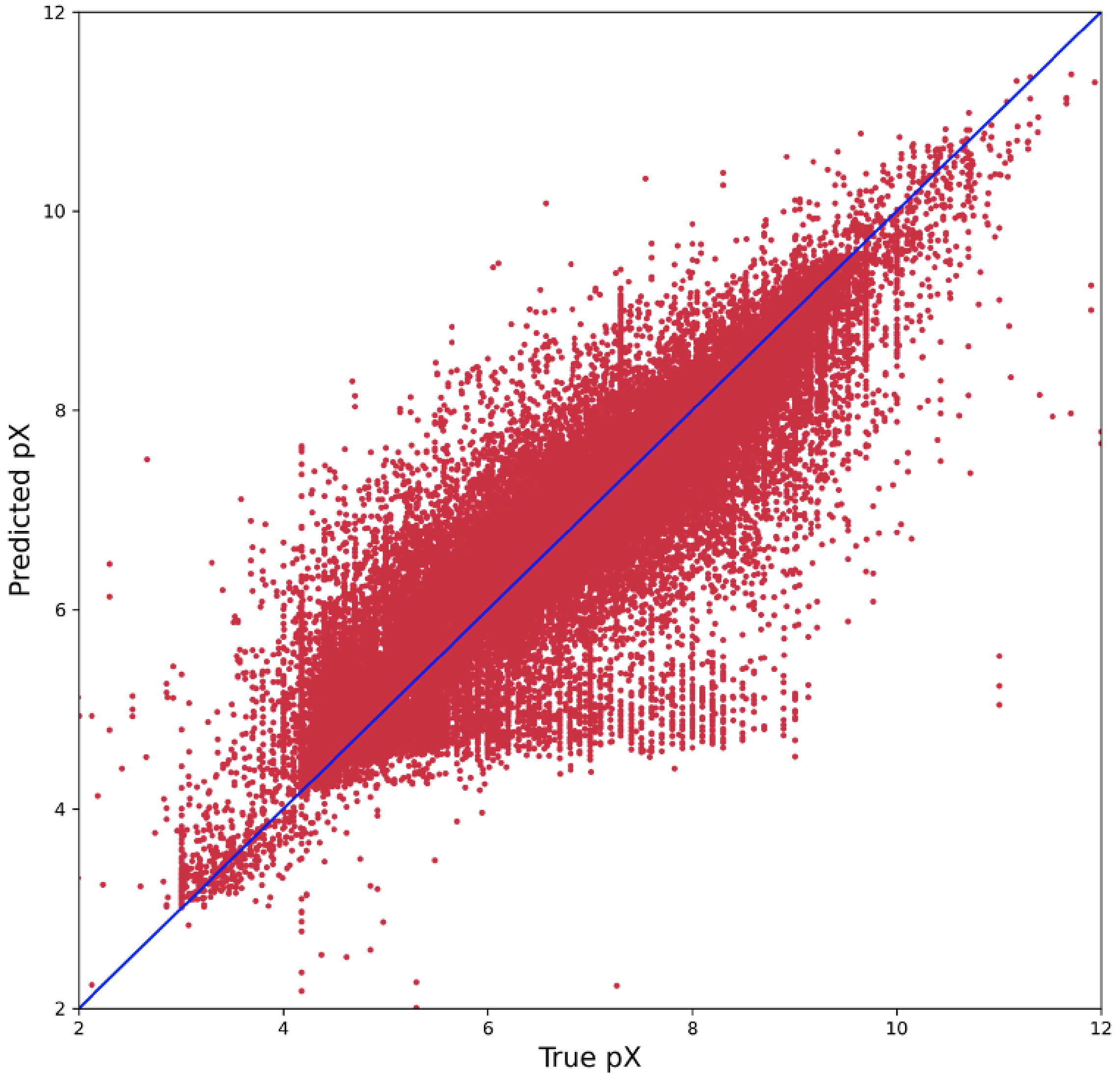

Figure S1: 5-fold cross-validation parity plot for Random Forest trained on Path molecular fingerprints (path length 5, fingerprint length 2048) with one-hot encoding used as protein descriptors for ABL kinase family. To evaluate the precision of experimental data on identical ligand-protein pairs, Kalliokoski et al. performed a reproducibility assessment using data from biochemical panels published in the literature[S6]. Their analysis revealed an average absolute error of 0.5 units in pIC_{50} measurements, which is consistent with the observed distribution of true pX to predicted pX matches from our machine learning models with mean absolute error of 0.51.

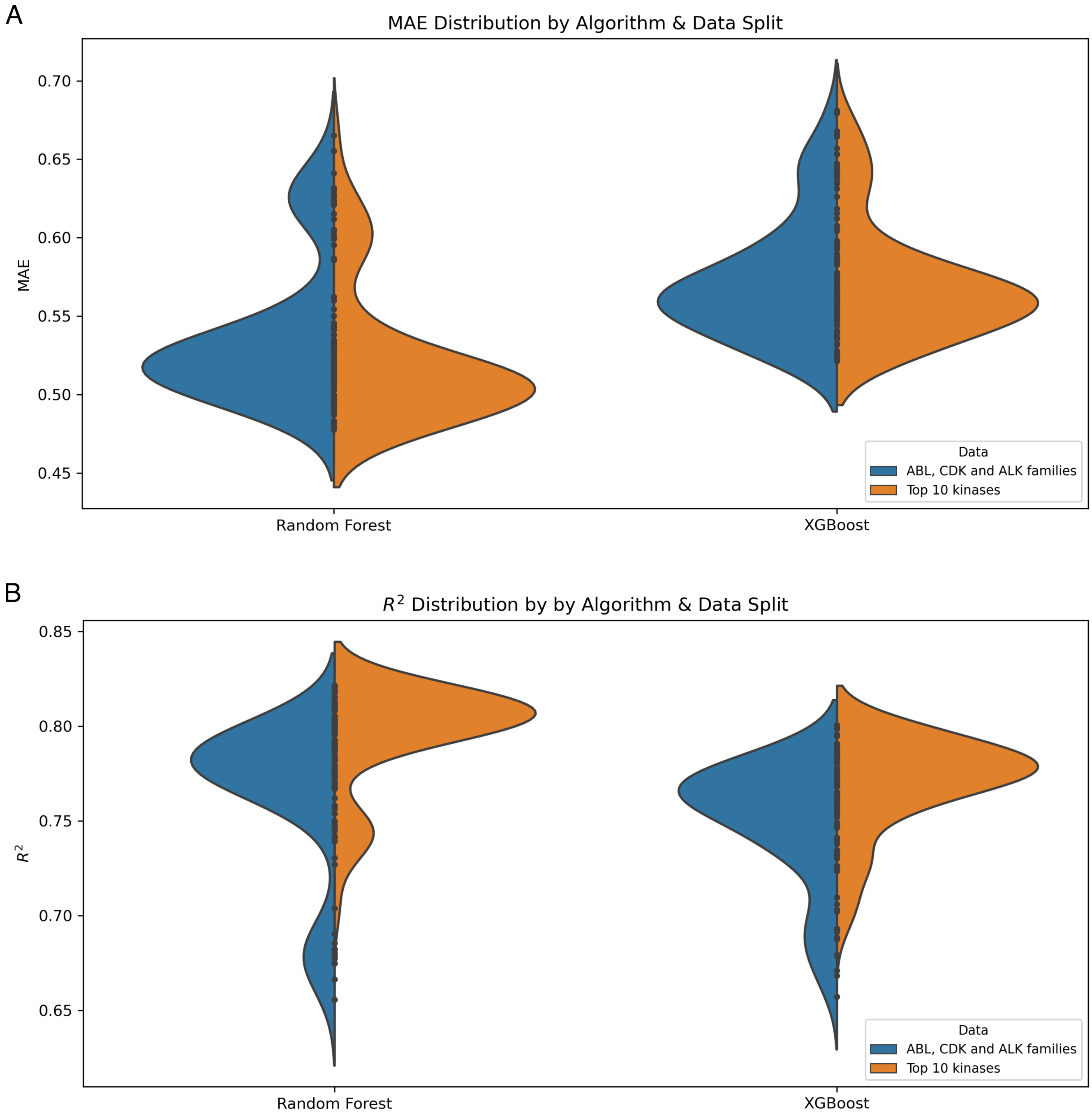

Figure S2: Violin plots of model performance across all 144 kinase–compound prediction models, trained with 5-fold cross-validation using Random Forest and XGBoost. (A) The distribution of mean absolute error (MAE); (B) The distribution of coefficient of determination (R^2). Each violin is split to compare two data-split scenarios—ABL,CDK, and ALK kinase families (blue) versus the top 10 most-represented kinases (orange), with the individual fold scores shown as points.

pendent kinase 12), TGFBR1 (TGF-beta receptor type-1), TGFBR2 (TGF-beta receptor type-2), ACVR2A (Activin receptor type-2A), ACVRL1 (Serine/threonine-protein kinase receptor R3), ACVR1 (Activin receptor type-1), ACVR2B (Activin receptor type-2B), BMPR1A (Bone morphogenetic protein receptor type-1A), ACVR1B (Activin receptor type-1B) and BMPR1B (Bone morphogenetic protein receptor type-1B).

For the top 10 represented human protein kinases, the selected kinases include MET (Hepatocyte growth factor receptor), EGFR (Epidermal growth factor receptor), JAK2 (Tyrosine protein kinase JAK2), MAPK14 (Mitogen-activated protein kinase 14), ABL1 (Tyrosine-protein kinase ABL1), AKT1 (RAC-alpha serine/threonine-protein kinase), MAPK1 (Mitogen-activated protein kinase 1), JAK3 (Tyrosine-protein kinase JAK3), SYK (Tyrosine-protein kinase SYK), and JAK1 (Tyrosine-protein kinase JAK1).

Table S1: Model performance on the ABL, CDK, and ALK kinase families across all 144 kinase–compound prediction models. Each model was trained using 5-fold cross-validation with either Random Forest or XGBoost. The *Model Label* encodes: (i) the type of Morgan fingerprint used, (ii) the fingerprint bit vector length, (iii) the fingerprint radius, and (iv) the type of amino acid descriptor employed.

**Model Label**	**MAE**	$\mathbf{R^2}$	**Model Type**
PATH 2048 5 OneHotEncoding	0.483	0.797	Random Forest
CIRC 1024 5 T-scales	0.533	0.778	Random Forest
CIRC 1024 5 Physical	0.532	0.779	Random Forest
PATH 512 3 Z-scale	0.532	0.771	Random Forest
PATH 1024 3 ST-scale	0.532	0.770	Random Forest
CIRC 512 3 Z-scale	0.530	0.779	Random Forest
CIRC 512 3 ST-scale	0.530	0.779	Random Forest
PATH 2048 3 ST-scale	0.535	0.767	Random Forest
PATH 512 3 T-scales	0.528	0.775	Random Forest
PATH 512 3 Physical	0.526	0.776	Random Forest

Continued on next page

Table S1 *Continued from previous page*

Model Label	MAE	R^2	Model Type
PATH 1024 3 Z-scale	0.526	0.775	Random Forest
CIRC 512 3 T-scales	0.524	0.783	Random Forest
PATH 2048 3 T-scales	0.524	0.778	Random Forest
PATH 1024 3 T-scales	0.524	0.778	Random Forest
PATH 512 5 ST-scale	0.523	0.782	Random Forest
PATH 2048 3 Z-scale	0.527	0.773	Random Forest
PATH 512 3 ST-scale	0.535	0.768	Random Forest
CIRC 512 3 Protein embedding	0.632	0.675	Random Forest
CIRC 2048 5 Protein embedding	0.630	0.675	Random Forest
CIRC 512 5 ST-scale	0.562	0.754	Random Forest
CIRC 512 5 Z-scale	0.560	0.757	Random Forest
CIRC 512 5 T-scales	0.555	0.762	Random Forest
PATH 1024 5 Protein embedding	0.612	0.691	Random Forest
CIRC 512 5 Physical	0.550	0.767	Random Forest
PATH 2048 5 Protein embedding	0.615	0.685	Random Forest
CIRC 2048 3 Protein embedding	0.622	0.680	Random Forest
PATH 1024 3 Protein embedding	0.622	0.682	Random Forest
PATH 512 5 Protein embedding	0.623	0.681	Random Forest
CIRC 512 5 OneHotEncoding	0.545	0.749	Random Forest
CIRC 1024 5 Z-scale	0.541	0.770	Random Forest
CIRC 1024 3 Protein embedding	0.623	0.682	Random Forest
PATH 2048 3 Protein embedding	0.628	0.677	Random Forest
CIRC 1024 5 ST-scale	0.538	0.771	Random Forest

Continued on next page

Table S1 *Continued from previous page*

Model Label	MAE	R^2	Model Type
PATH 512 3 Protein embedding	0.628	0.678	Random Forest
PATH 2048 3 Physical	0.522	0.778	Random Forest
PATH 1024 3 Physical	0.522	0.779	Random Forest
CIRC 1024 5 Protein embedding	0.641	0.666	Random Forest
CIRC 1024 3 Z-scale	0.521	0.782	Random Forest
CIRC 2048 5 Physical	0.510	0.793	Random Forest
PATH 2048 3 OneHotEncoding	0.510	0.778	Random Forest
CIRC 512 3 OneHotEncoding	0.509	0.778	Random Forest
CIRC 2048 3 ST-scale	0.509	0.790	Random Forest
PATH 1024 5 Z-scale	0.507	0.791	Random Forest
PATH 2048 5 ST-scale	0.503	0.793	Random Forest
PATH 512 5 OneHotEncoding	0.503	0.782	Random Forest
CIRC 512 5 Protein embedding	0.655	0.656	Random Forest
CIRC 2048 3 Z-scale	0.510	0.787	Random Forest
CIRC 2048 3 Physical	0.5	0.797	Random Forest
CIRC 2048 5 OneHotEncoding	0.499	0.787	Random Forest
CIRC 1024 3 OneHotEncoding	0.499	0.787	Random Forest
PATH 2048 5 Z-scale	0.498	0.796	Random Forest
PATH 1024 5 T-scales	0.498	0.8	Random Forest
PATH 2048 5 T-scales	0.491	0.803	Random Forest
PATH 1024 5 OneHotEncoding	0.489	0.792	Random Forest
PATH 2048 5 Physical	0.488	0.804	Random Forest
CIRC 2048 3 OneHotEncoding	0.487	0.796	Random Forest

Continued on next page

Table S1 *Continued from previous page*

Model Label	MAE	R^2	Model Type
PATH 1024 5 Physical	0.499	0.797	Random Forest
PATH 1024 5 ST-scale	0.511	0.789	Random Forest
CIRC 2048 3 T-scales	0.503	0.796	Random Forest
CIRC 1024 3 Physical	0.511	0.791	Random Forest
PATH 1024 3 OneHotEncoding	0.513	0.773	Random Forest
PATH 512 3 OneHotEncoding	0.521	0.768	Random Forest
CIRC 2048 5 T-scales	0.515	0.789	Random Forest
PATH 512 5 T-scales	0.515	0.788	Random Forest
CIRC 1024 3 T-scales	0.515	0.788	Random Forest
PATH 512 5 Z-scale	0.518	0.783	Random Forest
CIRC 512 3 Physical	0.519	0.788	Random Forest
CIRC 1024 5 OneHotEncoding	0.519	0.773	Random Forest
PATH 512 5 Physical	0.511	0.789	Random Forest
CIRC 2048 5 Z-scale	0.520	0.784	Random Forest
CIRC 2048 5 ST-scale	0.520	0.783	Random Forest
CIRC 1024 3 ST-scale	0.519	0.784	Random Forest
PATH 1024 3 Z-scale	0.586	0.740	XGBoost
PATH 1024 3 ST-scale	0.585	0.741	XGBoost
PATH 1024 3 T-scales	0.584	0.741	XGBoost
PATH 512 3 Physical	0.575	0.748	XGBoost
PATH 512 3 Z-scale	0.576	0.747	XGBoost
PATH 512 3 ST-scale	0.576	0.748	XGBoost
PATH 512 3 OneHotEncoding	0.587	0.738	XGBoost

Continued on next page

Table S1 *Continued from previous page*

**Model Label**	**MAE**	$\mathbf{R^2}$	**Model Type**
PATH 512 3 Protein embedding	0.653	0.679	XGBoost
PATH 512 3 T-scales	0.574	0.747	XGBoost
CIRC 512 5 OneHotEncoding	0.573	0.752	XGBoost
CIRC 2048 5 OneHotEncoding	0.572	0.755	XGBoost
PATH 1024 3 Physical	0.583	0.741	XGBoost
PATH 2048 3 Physical	0.593	0.735	XGBoost
PATH 2048 3 OneHotEncoding	0.604	0.726	XGBoost
PATH 2048 3 ST-scale	0.596	0.732	XGBoost
PATH 1024 3 OneHotEncoding	0.598	0.731	XGBoost
PATH 2048 3 T-scales	0.598	0.730	XGBoost
CIRC 512 5 Protein embedding	0.653	0.671	XGBoost
PATH 1024 5 Protein embedding	0.612	0.706	XGBoost
PATH 512 5 Protein embedding	0.616	0.703	XGBoost
PATH 2048 5 Protein embedding	0.618	0.702	XGBoost
CIRC 1024 5 Protein embedding	0.647	0.679	XGBoost
CIRC 2048 5 Protein embedding	0.644	0.688	XGBoost
CIRC 2048 3 Protein embedding	0.642	0.693	XGBoost
CIRC 1024 5 OneHotEncoding	0.570	0.754	XGBoost
CIRC 1024 3 Protein embedding	0.638	0.691	XGBoost
CIRC 512 3 Protein embedding	0.636	0.688	XGBoost
PATH 2048 3 Z-scale	0.595	0.732	XGBoost
CIRC 2048 3 OneHotEncoding	0.569	0.759	XGBoost
CIRC 512 3 Physical	0.547	0.772	XGBoost

Continued on next page

Table S1 *Continued from previous page*

Model Label	MAE	R^2	Model Type
CIRC 512 5 ST-scale	0.567	0.757	XGBoost
CIRC 512 3 Z-scale	0.548	0.772	XGBoost
PATH 1024 3 Protein embedding	0.668	0.668	XGBoost
PATH 512 5 OneHotEncoding	0.547	0.769	XGBoost
PATH 512 5 Z-scale	0.539	0.774	XGBoost
PATH 512 5 Physical	0.539	0.774	XGBoost
PATH 2048 5 OneHotEncoding	0.537	0.777	XGBoost
PATH 1024 5 OneHotEncoding	0.536	0.776	XGBoost
PATH 512 5 T-scales	0.536	0.776	XGBoost
PATH 512 5 ST-scale	0.535	0.776	XGBoost
PATH 2048 5 ST-scale	0.528	0.782	XGBoost
PATH 1024 5 Physical	0.527	0.781	XGBoost
PATH 1024 5 ST-scale	0.525	0.783	XGBoost
PATH 2048 5 T-scales	0.525	0.783	XGBoost
PATH 2048 5 Z-scale	0.525	0.785	XGBoost
PATH 2048 5 Physical	0.523	0.786	XGBoost
PATH 1024 5 Z-scale	0.522	0.785	XGBoost
PATH 1024 5 T-scales	0.521	0.786	XGBoost
CIRC 512 3 T-scales	0.549	0.772	XGBoost
CIRC 1024 3 T-scales	0.549	0.771	XGBoost
CIRC 1024 3 Z-scale	0.550	0.772	XGBoost
CIRC 1024 3 ST-scale	0.550	0.770	XGBoost
CIRC 512 5 Physical	0.565	0.760	XGBoost

Continued on next page

Table S1 *Continued from previous page*

Model Label	MAE	R^2	Model Type
CIRC 1024 5 Z-scale	0.564	0.759	XGBoost
CIRC 2048 5 ST-scale	0.564	0.761	XGBoost
CIRC 512 5 Z-scale	0.562	0.761	XGBoost
CIRC 2048 5 Physical	0.562	0.763	XGBoost
CIRC 1024 3 OneHotEncoding	0.561	0.762	XGBoost
CIRC 1024 5 Physical	0.561	0.763	XGBoost
CIRC 2048 3 ST-scale	0.561	0.764	XGBoost
CIRC 512 5 T-scales	0.567	0.758	XGBoost
CIRC 2048 5 Z-scale	0.561	0.765	XGBoost
CIRC 2048 5 T-scales	0.559	0.765	XGBoost
CIRC 1024 5 ST-scale	0.559	0.762	XGBoost
CIRC 1024 5 T-scales	0.559	0.764	XGBoost
CIRC 2048 3 Z-scale	0.558	0.765	XGBoost
CIRC 2048 3 T-scales	0.556	0.768	XGBoost
CIRC 2048 3 Physical	0.555	0.769	XGBoost
CIRC 1024 3 Physical	0.550	0.771	XGBoost
CIRC 512 3 ST-scale	0.550	0.771	XGBoost
CIRC 512 3 OneHotEncoding	0.560	0.763	XGBoost
PATH 2048 3 Protein embedding	0.681	0.657	XGBoost

Table S2: Model performance on the top 10 kinases across all 144 kinase–compound prediction models. Each model was trained using 5-fold cross-validation with either Random Forest or XGBoost. The *Model Label* encodes: (i) the type of Morgan fingerprint used, (ii) the fingerprint bit vector length, (iii) the fingerprint radius, and (iv) the type of amino acid descriptor employed.

**Model Label**	**MAE**	**R^2**	**Model Type**
CIRC 2048 3 OneHotEncoding	0.478	0.818	Random Forest
PATH 512 3 Z-scale	0.517	0.799	Random Forest
CIRC 1024 5 ST-scale	0.516	0.799	Random Forest
PATH 512 3 Physical	0.516	0.799	Random Forest
CIRC 1024 5 Z-scale	0.515	0.799	Random Forest
CIRC 1024 5 T-scales	0.515	0.800	Random Forest
CIRC 1024 5 Physical	0.515	0.800	Random Forest
PATH 512 3 T-scales	0.517	0.799	Random Forest
PATH 512 3 OneHotEncoding	0.513	0.800	Random Forest
PATH 1024 3 Physical	0.511	0.801	Random Forest
PATH 1024 3 Z-scale	0.511	0.801	Random Forest
PATH 1024 3 T-scales	0.511	0.801	Random Forest
CIRC 512 3 OneHotEncoding	0.511	0.802	Random Forest
CIRC 512 3 ST-scale	0.510	0.805	Random Forest
CIRC 512 3 Physical	0.510	0.805	Random Forest
PATH 1024 3 ST-scale	0.512	0.801	Random Forest
PATH 512 3 ST-scale	0.517	0.799	Random Forest
CIRC 1024 5 OneHotEncoding	0.517	0.797	Random Forest
CIRC 512 5 Physical	0.542	0.788	Random Forest
CIRC 512 5 Protein embedding	0.665	0.704	Random Forest
CIRC 1024 5 Protein embedding	0.627	0.727	Random Forest

Continued on next page

Table S2 *Continued from previous page*

Model Label	MAE	R^2	Model Type
CIRC 512 3 Protein embedding	0.626	0.731	Random Forest
PATH 512 5 Protein embedding	0.621	0.739	Random Forest
PATH 512 3 Protein embedding	0.605	0.742	Random Forest
PATH 1024 5 Protein embedding	0.604	0.748	Random Forest
CIRC 2048 5 Protein embedding	0.602	0.741	Random Forest
CIRC 1024 3 Protein embedding	0.600	0.745	Random Forest
PATH 1024 3 Protein embedding	0.599	0.745	Random Forest
PATH 2048 3 Protein embedding	0.595	0.747	Random Forest
PATH 2048 5 Protein embedding	0.587	0.758	Random Forest
CIRC 2048 3 Protein embedding	0.586	0.750	Random Forest
CIRC 512 5 OneHotEncoding	0.544	0.784	Random Forest
CIRC 512 5 T-scales	0.543	0.787	Random Forest
CIRC 512 5 Z-scale	0.542	0.788	Random Forest
CIRC 512 3 T-scales	0.510	0.805	Random Forest
CIRC 512 3 Z-scale	0.510	0.806	Random Forest
CIRC 512 5 ST-scale	0.543	0.787	Random Forest
PATH 2048 3 ST-scale	0.509	0.801	Random Forest
CIRC 2048 3 ST-scale	0.479	0.818	Random Forest
CIRC 2048 3 Physical	0.480	0.818	Random Forest
PATH 1024 5 T-scales	0.495	0.816	Random Forest
CIRC 2048 5 Z-scale	0.495	0.811	Random Forest
PATH 2048 5 ST-scale	0.482	0.822	Random Forest
CIRC 1024 3 ST-scale	0.494	0.812	Random Forest

Continued on next page

Table S2 *Continued from previous page*

Model Label	MAE	R^2	Model Type
CIRC 2048 3 T-scales	0.479	0.818	Random Forest
PATH 2048 5 OneHotEncoding	0.482	0.820	Random Forest
PATH 2048 5 Physical	0.482	0.821	Random Forest
PATH 2048 5 Z-scale	0.483	0.821	Random Forest
CIRC 1024 3 T-scales	0.493	0.813	Random Forest
CIRC 1024 3 Physical	0.493	0.813	Random Forest
CIRC 1024 3 Z-scale	0.494	0.812	Random Forest
PATH 2048 3 T-scales	0.509	0.801	Random Forest
PATH 2048 5 T-scales	0.482	0.821	Random Forest
CIRC 2048 3 Z-scale	0.478	0.819	Random Forest
PATH 1024 5 Physical	0.495	0.816	Random Forest
CIRC 2048 5 OneHotEncoding	0.495	0.809	Random Forest
PATH 2048 3 Z-scale	0.508	0.801	Random Forest
PATH 2048 3 Physical	0.508	0.802	Random Forest
PATH 1024 3 OneHotEncoding	0.508	0.802	Random Forest
PATH 512 5 Physical	0.507	0.809	Random Forest
CIRC 2048 5 ST-scale	0.495	0.810	Random Forest
PATH 512 5 Z-scale	0.507	0.809	Random Forest
PATH 512 5 ST-scale	0.507	0.810	Random Forest
PATH 512 5 OneHotEncoding	0.506	0.808	Random Forest
PATH 2048 3 OneHotEncoding	0.505	0.802	Random Forest
PATH 1024 5 OneHotEncoding	0.496	0.813	Random Forest
CIRC 2048 5 T-scales	0.496	0.810	Random Forest

Continued on next page

Table S2 *Continued from previous page*

**Model Label**	**MAE**	**R^2**	**Model Type**
CIRC 2048 5 Physical	0.496	0.810	Random Forest
PATH 512 5 T-scales	0.507	0.809	Random Forest
PATH 1024 5 Z-scale	0.496	0.815	Random Forest
PATH 1024 5 ST-scale	0.496	0.815	Random Forest
CIRC 1024 3 OneHotEncoding	0.495	0.810	Random Forest
PATH 1024 5 Protein embedding	0.608	0.747	XGBoost
PATH 1024 3 ST-scale	0.578	0.763	XGBoost
PATH 1024 3 Physical	0.577	0.764	XGBoost
CIRC 512 5 Protein embedding	0.664	0.710	XGBoost
PATH 1024 3 Z-scale	0.577	0.764	XGBoost
CIRC 512 5 OneHotEncoding	0.575	0.768	XGBoost
CIRC 512 5 T-scales	0.569	0.771	XGBoost
PATH 1024 3 T-scales	0.578	0.764	XGBoost
CIRC 512 5 Z-scale	0.568	0.772	XGBoost
CIRC 2048 5 OneHotEncoding	0.568	0.772	XGBoost
CIRC 512 5 ST-scale	0.568	0.771	XGBoost
CIRC 512 5 Physical	0.568	0.771	XGBoost
PATH 512 3 OneHotEncoding	0.572	0.768	XGBoost
PATH 1024 3 OneHotEncoding	0.583	0.760	XGBoost
PATH 512 3 Protein embedding	0.657	0.710	XGBoost
CIRC 1024 5 OneHotEncoding	0.567	0.773	XGBoost
CIRC 1024 5 Protein embedding	0.645	0.724	XGBoost
PATH 2048 3 Physical	0.587	0.756	XGBoost

Continued on next page

Table S2 *Continued from previous page*

Model Label	MAE	R^2	Model Type
PATH 2048 3 T-scales	0.588	0.756	XGBoost
PATH 2048 3 ST-scale	0.590	0.755	XGBoost
PATH 2048 3 OneHotEncoding	0.593	0.753	XGBoost
CIRC 2048 5 Protein embedding	0.644	0.725	XGBoost
CIRC 512 3 Protein embedding	0.640	0.726	XGBoost
CIRC 2048 3 Protein embedding	0.635	0.732	XGBoost
CIRC 1024 3 Protein embedding	0.631	0.734	XGBoost
PATH 512 5 Protein embedding	0.626	0.733	XGBoost
PATH 2048 5 Protein embedding	0.605	0.749	XGBoost
PATH 2048 3 Z-scale	0.586	0.758	XGBoost
PATH 512 3 T-scales	0.566	0.771	XGBoost
CIRC 2048 5 Z-scale	0.559	0.777	XGBoost
PATH 512 3 Physical	0.565	0.772	XGBoost
CIRC 1024 3 Z-scale	0.549	0.786	XGBoost
PATH 512 5 OneHotEncoding	0.545	0.788	XGBoost
PATH 512 5 T-scales	0.544	0.788	XGBoost
PATH 1024 3 Protein embedding	0.666	0.703	XGBoost
PATH 512 5 ST-scale	0.541	0.789	XGBoost
PATH 512 5 Z-scale	0.541	0.789	XGBoost
PATH 512 5 Physical	0.540	0.791	XGBoost
PATH 1024 5 OneHotEncoding	0.533	0.796	XGBoost
PATH 2048 5 OneHotEncoding	0.532	0.795	XGBoost
PATH 1024 5 T-scales	0.528	0.799	XGBoost

Continued on next page

Table S2 *Continued from previous page*

**Model Label**	**MAE**	**R^2**	**Model Type**
PATH 2048 5 T-scales	0.527	0.799	XGBoost
PATH 1024 5 Physical	0.527	0.799	XGBoost
PATH 1024 5 ST-scale	0.526	0.799	XGBoost
PATH 2048 5 Physical	0.526	0.799	XGBoost
PATH 2048 5 ST-scale	0.525	0.799	XGBoost
PATH 1024 5 Z-scale	0.525	0.801	XGBoost
PATH 2048 5 Z-scale	0.525	0.799	XGBoost
CIRC 512 3 Z-scale	0.549	0.785	XGBoost
CIRC 1024 3 Physical	0.549	0.785	XGBoost
CIRC 512 3 Physical	0.549	0.785	XGBoost
CIRC 1024 3 T-scales	0.549	0.784	XGBoost
PATH 512 3 Z-scale	0.565	0.773	XGBoost
CIRC 2048 3 OneHotEncoding	0.562	0.778	XGBoost
CIRC 2048 5 ST-scale	0.562	0.776	XGBoost
CIRC 2048 5 T-scales	0.561	0.776	XGBoost
CIRC 2048 5 Physical	0.560	0.777	XGBoost
CIRC 1024 5 T-scales	0.560	0.777	XGBoost
CIRC 1024 5 ST-scale	0.559	0.777	XGBoost
CIRC 1024 5 Z-scale	0.559	0.777	XGBoost
PATH 512 3 ST-scale	0.566	0.771	XGBoost
CIRC 1024 5 Physical	0.557	0.778	XGBoost
CIRC 2048 3 Physical	0.555	0.780	XGBoost
CIRC 512 3 OneHotEncoding	0.554	0.782	XGBoost

Continued on next page

Table S2 *Continued from previous page*

Model Label	MAE	R^2	Model Type
CIRC 2048 3 ST-scale	0.554	0.782	XGBoost
CIRC 2048 3 T-scales	0.553	0.783	XGBoost
CIRC 2048 3 Z-scale	0.553	0.783	XGBoost
CIRC 512 3 ST-scale	0.551	0.783	XGBoost
CIRC 512 3 T-scales	0.551	0.783	XGBoost
CIRC 1024 3 ST-scale	0.551	0.783	XGBoost
CIRC 1024 3 OneHotEncoding	0.555	0.781	XGBoost
PATH 2048 3 Protein embedding	0.680	0.693	XGBoost

SI-1.3 Analysis of embeddings

The overlaps between close neighbors representing different kinase families on the UMAP illustrate complex kinase evolutionary relationships. We selected two representative cases of kinases that were in close proximity on the UMAP of AlphaFold2 embeddings, spanning both intra-family and inter-family groupings, for detailed structural and sequence comparisons (Supp. Figure S3). For instance, we compared two CAMK kinases, A and G, with another kinase, IϰBKβ, as shown on Supp. Figure S3A. Despite their embedding proximity illustrated on the UMAP, the structural alignment revealed minimal differences, except for an extended β-sheet present in IϰBKβ. In terms of sequence similarity, CAMK kinases A and G share a high similarity of 95% with each other but only 45% with IϰBKβ, illustrating that the AlphaFold2 embedding representations capture structural similarities rather than sequence similarities. This pattern does not hold for ESM2 embeddings, where the proximity strictly indicates closeness within kinase families rather than across them.

Next, two TKL kinases (RIPK1 and LIMK2) were compared with two TYR kinases

(CSK and TXK) selected due to RIPK1's proximity to the TYR kinases, as illustrated on the UMAP, unlike LIMK2. This offered a test as to whether kinases are grouped more by structural or by sequence similarities. The structures of RIPK1, CSK, and TXK demonstrated substantial structural alignment, setting LIMK2 apart due to a disordered region extending from an α-helix. Interestingly, the two TYR kinases shared 65% sequence similarity, while the other kinase pairs exhibited only about 50% (Supp. Figure S3B), suggesting that the AlphaFold2 embeddings illustrated on the UMAP representation reflects structural features more than sequence similarity. However, in contrast with AlphaFold2 embeddings, the ESM2 embeddings emphasized family-based groupings, suggesting that they may prioritize sequence similarity or familial affiliations over structural nuances. These observations indicate that the AlphaFold2 embedding space is modulated by structural similarities, whereas the ESM2 embedding space is influenced by sequence similarity and familial relationships.

SI-1.4 Hyperparameter tuning

Hyperparameter tuning was conducted using Optuna[S7] through a nested cross-validation. For each data splitting strategy, datasets were divided into five folds for random and kinase splits, and three folds for family splits. Each round of cross-validation involved hyperparameter optimization over 100 trials. During each trial, a secondary 5-fold cross-validation, mirroring the primary data splitting method, was employed, with the exception for the family split, where a kinase split was used for the inner validations. The average metric from the inner cross-validation loop served as the endpoint for each trial, guiding the hyperparameter space exploration towards maximization goals. The optimal set of parameters identified was then validated in the outer cross-validation loop. The parameters that yielded the best-performing model across all outer loops were selected to train the final model, which was subsequently applied in all permutation experiments.

For the XGBoost models, the optimized parameters included `max_depth`, `max_leaves`, `n_estimators`, `gamma`, `early_stopping_rounds`, `reg_lambda`, `reg_alpha`, and `learning_rate`.

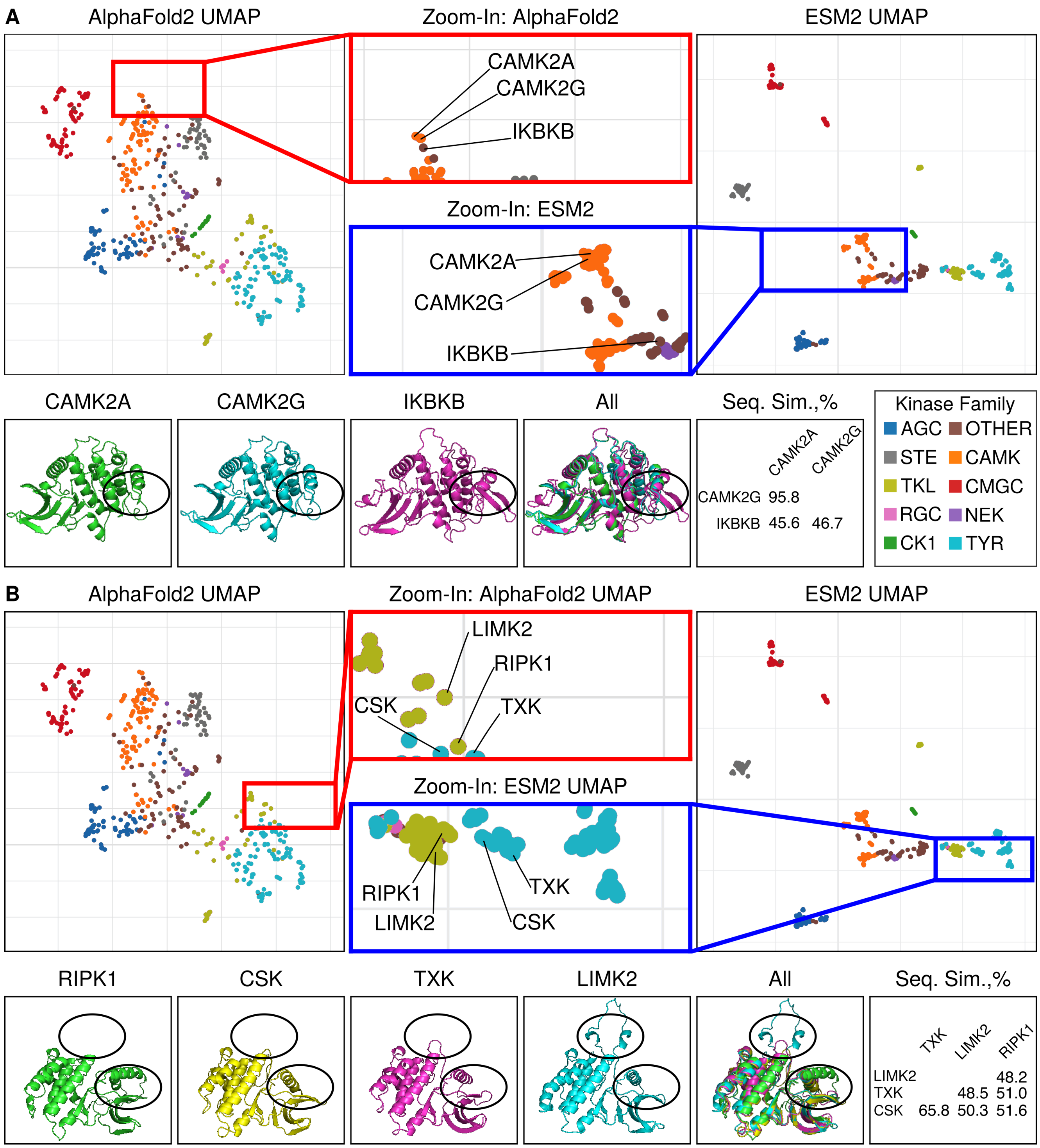

Figure S3: Pairwise analysis of kinase domain structures illustrated by UMAP projections of protein embeddings. (A) Grouping of CAMK2A, CAMK2G, and IκBKβ on the AlphaFold2 and ESM2 UMAPs (top), with a detailed comparison of their sequence and structural similarities (bottom). (B) Grouping of RIPK1, LIMK2, TXK and CSK kinases on the AlphaFold2 and ESM2 UMAPs (top) with the structural and sequence similarities analysis (bottom). Significant structural differences are highlighted with black ellipses.

Models were trained targeting a binary logistic objective, with the area under the curve serving as the primary evaluation metric.

SI-1.5 Implementation of Convolutional Autoencoder

The convolutional autoencoder models were trained over 100 epochs, utilizing 80% of the embeddings for training and 20% for validation. To enhance the model's robustness, data augmentation was employed by adding Gaussian noise into a random subset of the embeddings. This noise was drawn from a standard normal distribution with a standard deviation of 0.3, effectively tripling the size of the training dataset. A learning rate of 1e-5 was set, and adaptive adjustments were made using the ReduceLROnPlateau scheduler to optimize the learning rate during training. Upon training completion, the models were applied to the entire set of embeddings.

Table S3: The architecture of CAEs used to reduce the dimensions of 2D embeddings

		Embedding Model	
		**AF2**	**ESM2**
**Input Size**		227×384	227×1280
**Resized input**		256×384	256×1280
**Convolution**	**Kernel**	9	10
	**Padding**	4	3
	**Stride**	1	1
**Max-Pool**	**Kernel**	4	4
	**Padding**	0	1
	**Stride**	4	2
**No. of blocks**		3	4
**Latent Dimension**		192	1680
**Activation**		ReLU	LeakyReLU
**Optimizer**		RMSprop (momentum = 0.1)	Adam

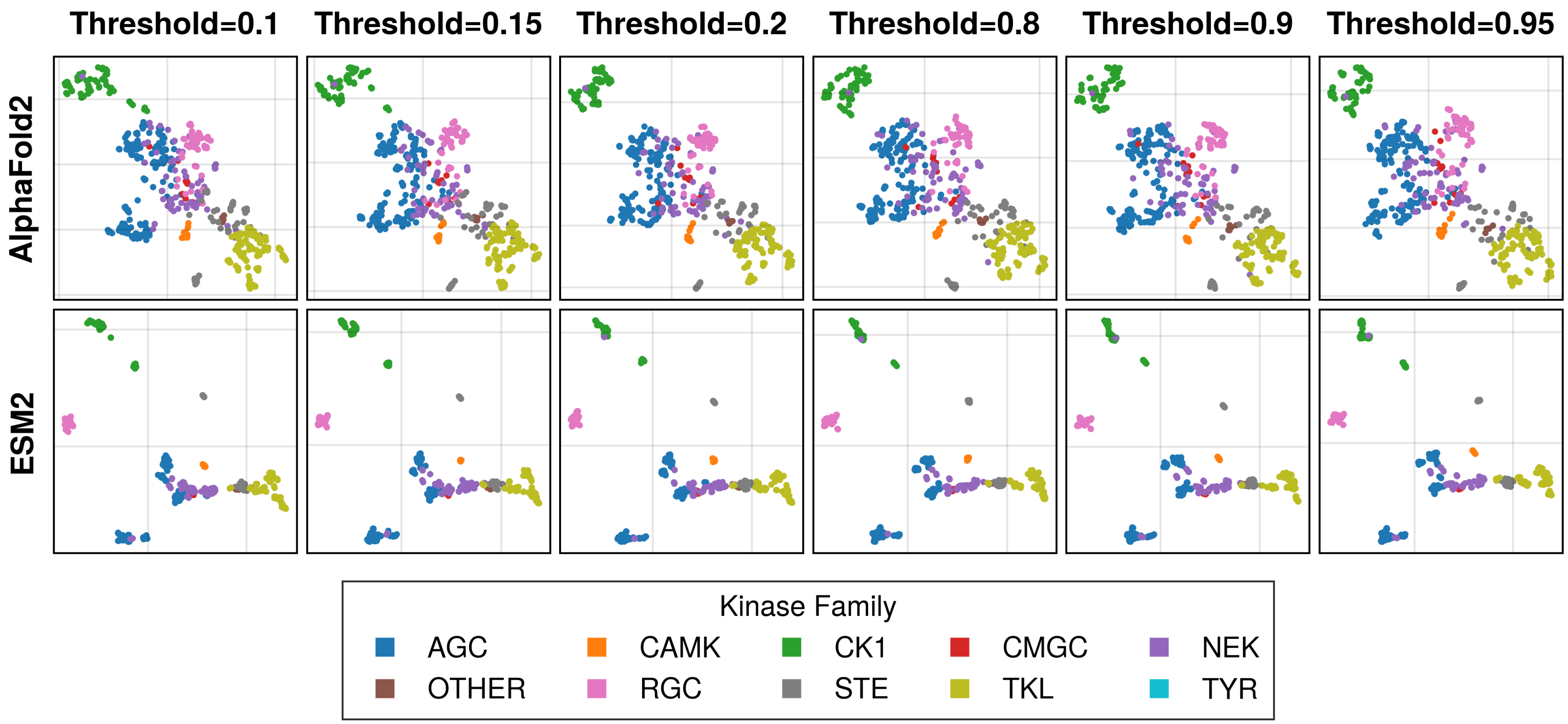

Figure S4: UMAP projections of ESM2 and AlphaFold2 structural embeddings with varied trimming thresholds. The thresholds are set based on the percentage of sequences in MSA that display a gap at a particular position, with a higher threshold leading to more extensive pruning of the embeddings. Despite the variations in thresholds, the clustering patterns remain consistent, indicating robustness across different levels of sparsity reduction. Consequently, a threshold of 95% was selected to minimize sparsity while preserving structural detail.

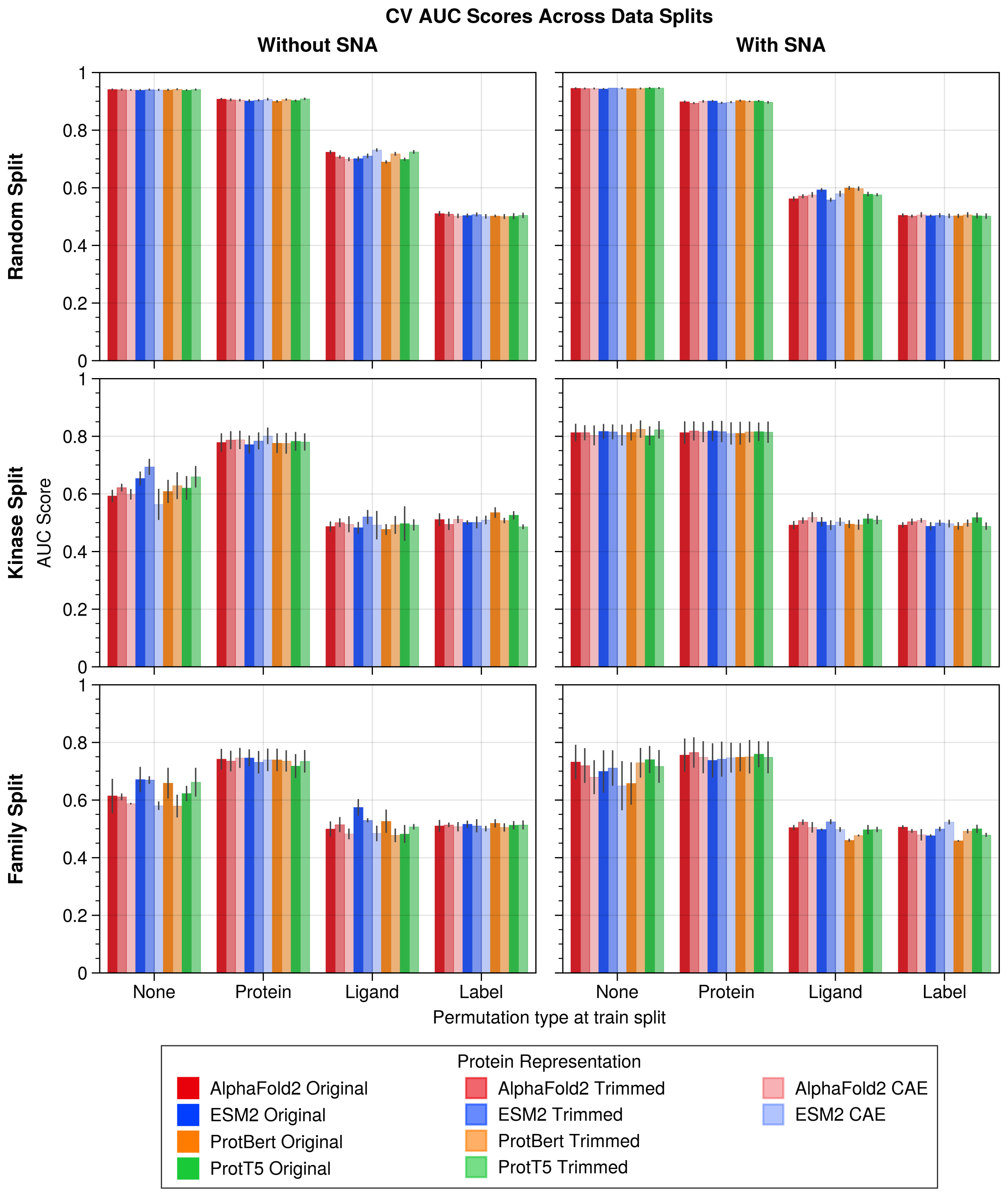

Figure S5: Performance of XGBoost models trained to predict bioactivity of kinase-ligand pairs as measured by AUROC (mean ± s.e.) across cross-validation folds. Different cross-validation splits were created using either random split (top row), kinase split (middle row) or family split (bottom row) strategies. Models were trained with the dataset as is (left column) or after rebalancing with SNA (right column) using a ratio of 1:1 inactives to actives. Embeddings or labels were also permuted during training (depicted on X-axis) and compared with the baseline (no permutation; "None").

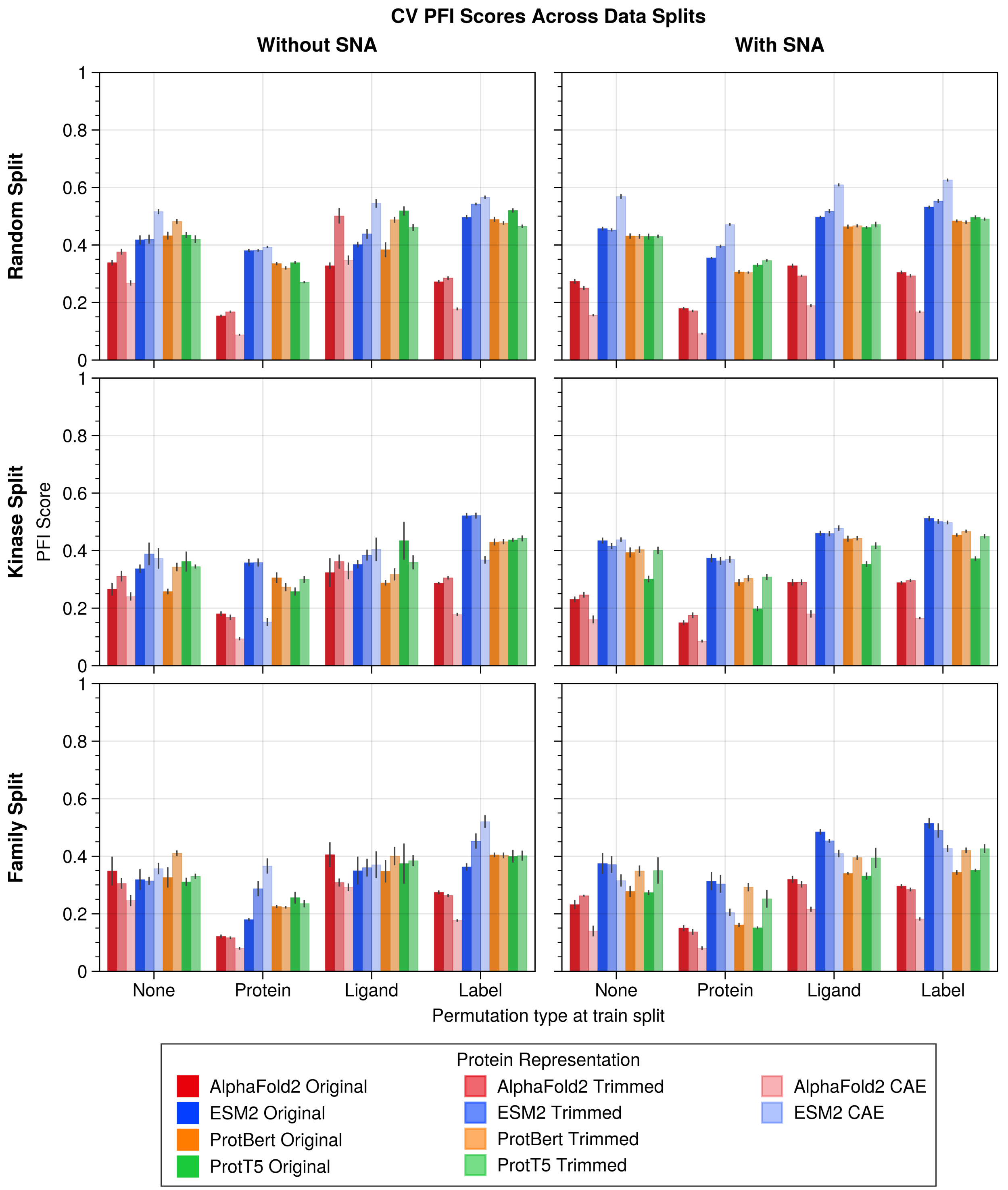

Figure S6: Performance of XGBoost models trained to predict bioactivity of kinase-ligand pairs as measured by PFI (mean ± s.e.) across cross-validation folds. Different cross-validation splits were created using either random split (top row), kinase split (middle row) or family split (bottom row) strategies. Models were trained with the dataset as is (left column) or after rebalancing with SNA (right column) using a ratio of 1:1 inactives to actives. Embeddings or labels were also permuted during training (depicted on X-axis) and compared with the baseline (no permutation; "None").

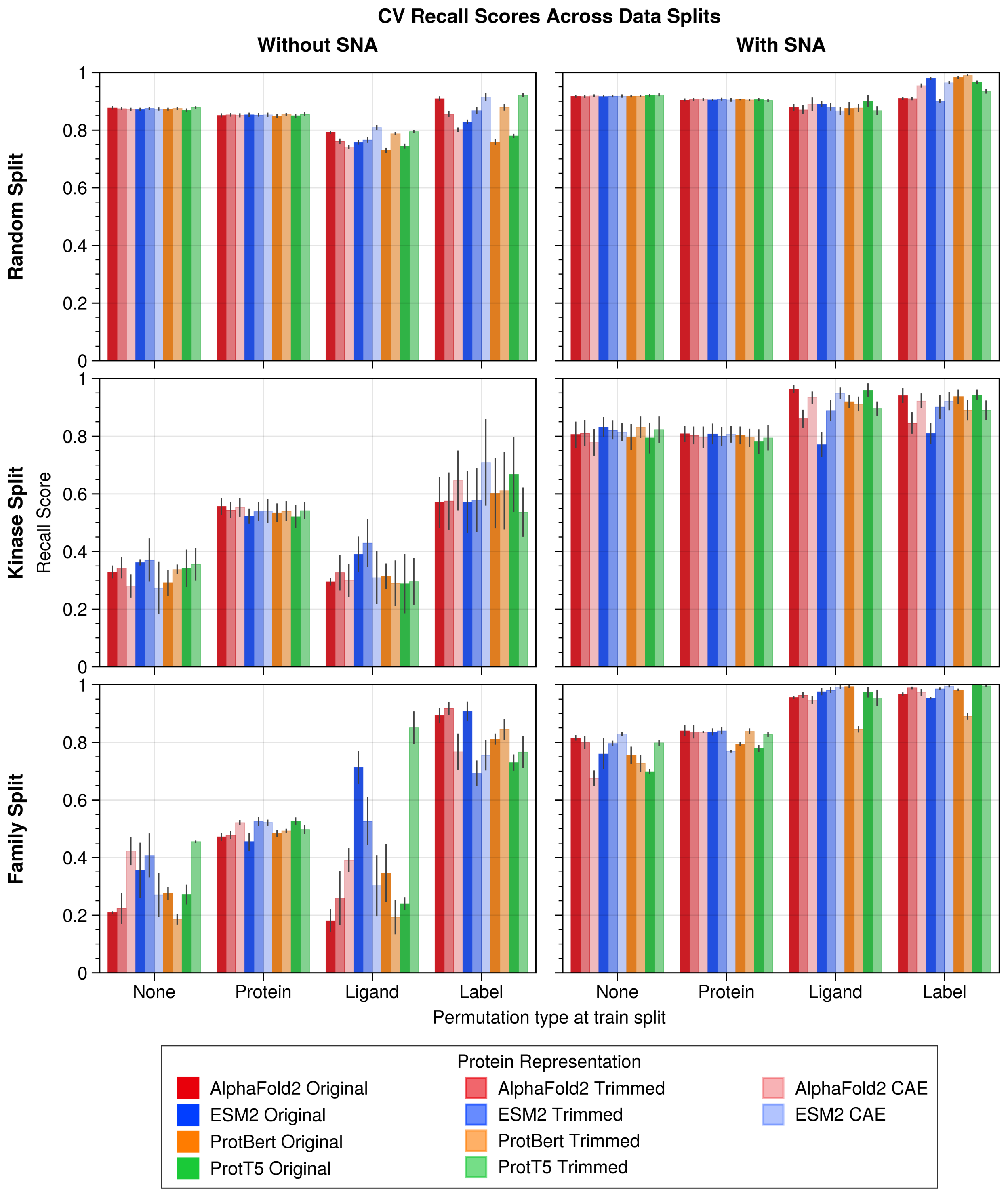

Figure S7: Performance of XGBoost models trained to predict bioactivity of kinase-ligand pairs as measured by Recall (mean ± s.e.) across cross-validation folds. Different cross-validation splits were created using either random split (top row), kinase split (middle row) or family split (bottom row) strategies. Models were trained with the dataset as is (left column) or after rebalancing with SNA (right column) using a ratio of 1:1 inactives to actives. Embeddings or labels were also permuted during training (depicted on X-axis) and compared with the baseline (no permutation; "None").

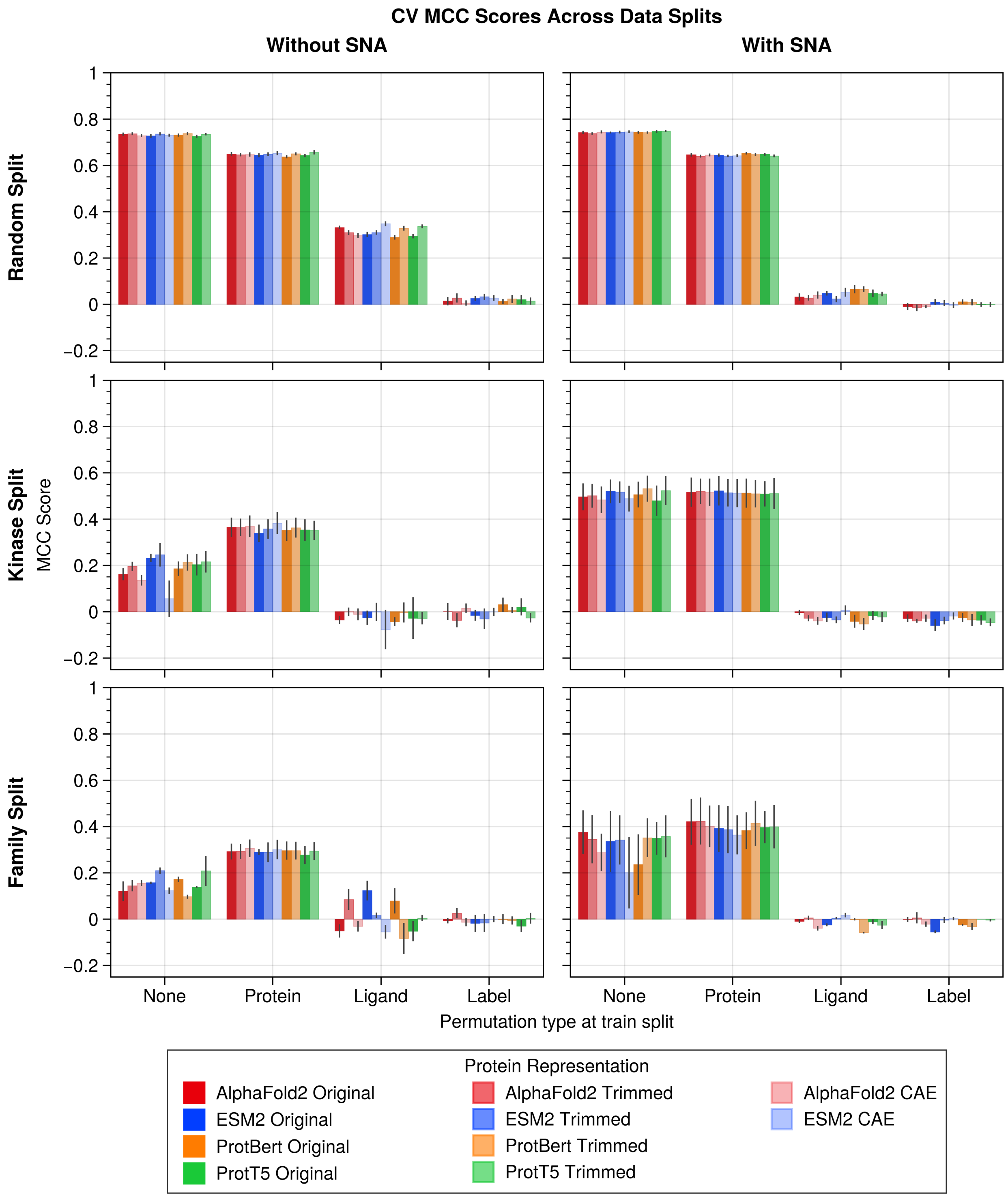

Figure S8: Performance of XGBoost models trained to predict bioactivity of kinase-ligand pairs as measured by MCC (mean ± s.e.) across cross-validation folds with permutations on the train set. Different cross-validation splits were created using either random split (top row), kinase split (middle row) or family split (bottom row) strategies. Models were trained with the dataset as is (left column) or after rebalancing with SNA (right column) using a ratio of 1:1 inactives to actives. Embeddings or labels were also permuted during training (depicted on X-axis) and compared with the baseline (no permutation; "None").

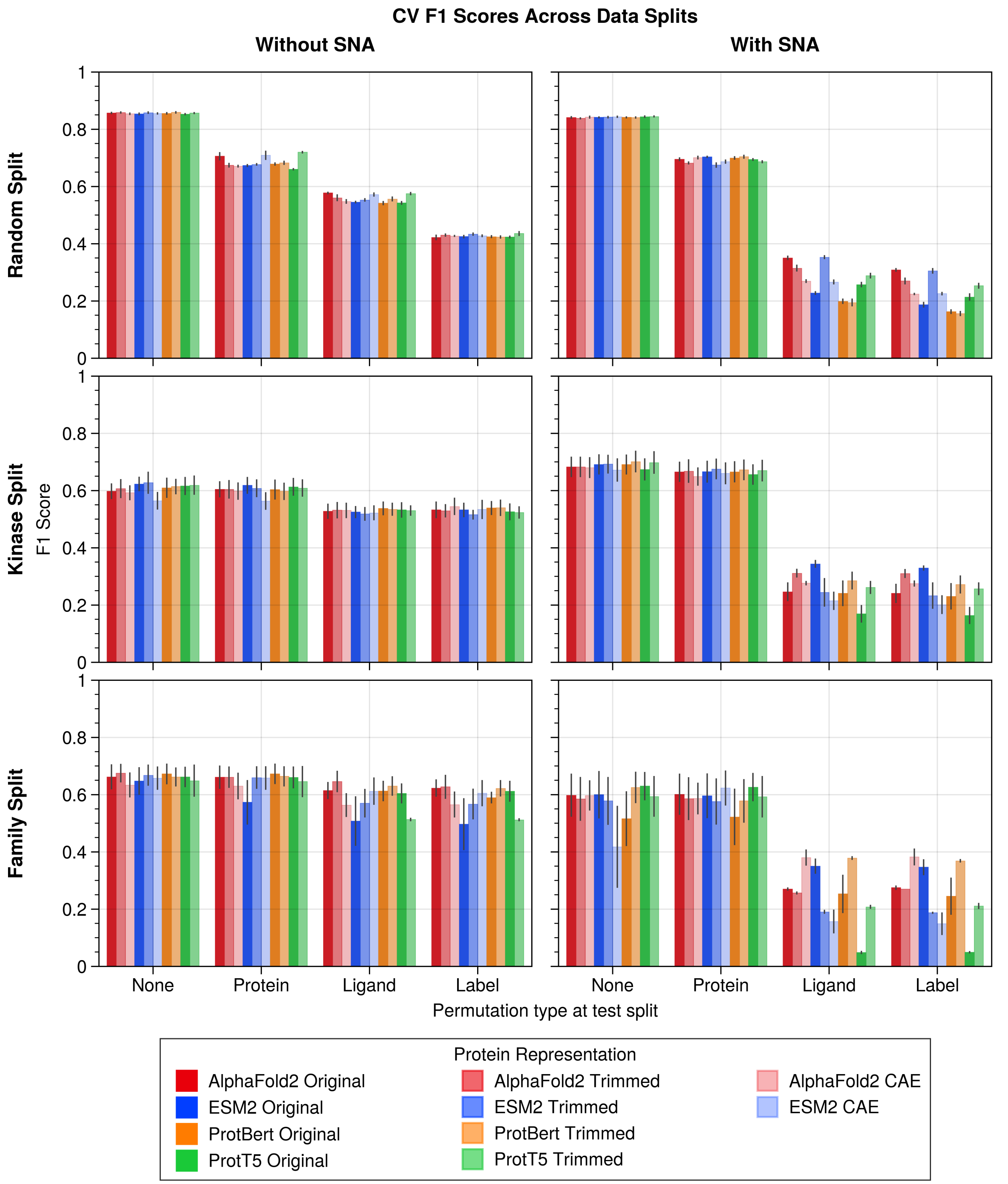

Figure S9: Performance of XGBoost models trained to predict bioactivity of kinase-ligand pairs as measured by F1 score (mean ± s.e.) across cross-validation folds with permutations on the test set and compared with the baseline (no permutation; "None"). Different cross-validation splits were created using either random split (top row), kinase split (middle row) or family split (bottom row) strategies. Models were trained with the dataset as is (left column) or after rebalancing with SNA (right column) using a ratio of 1:1 inactives to actives.

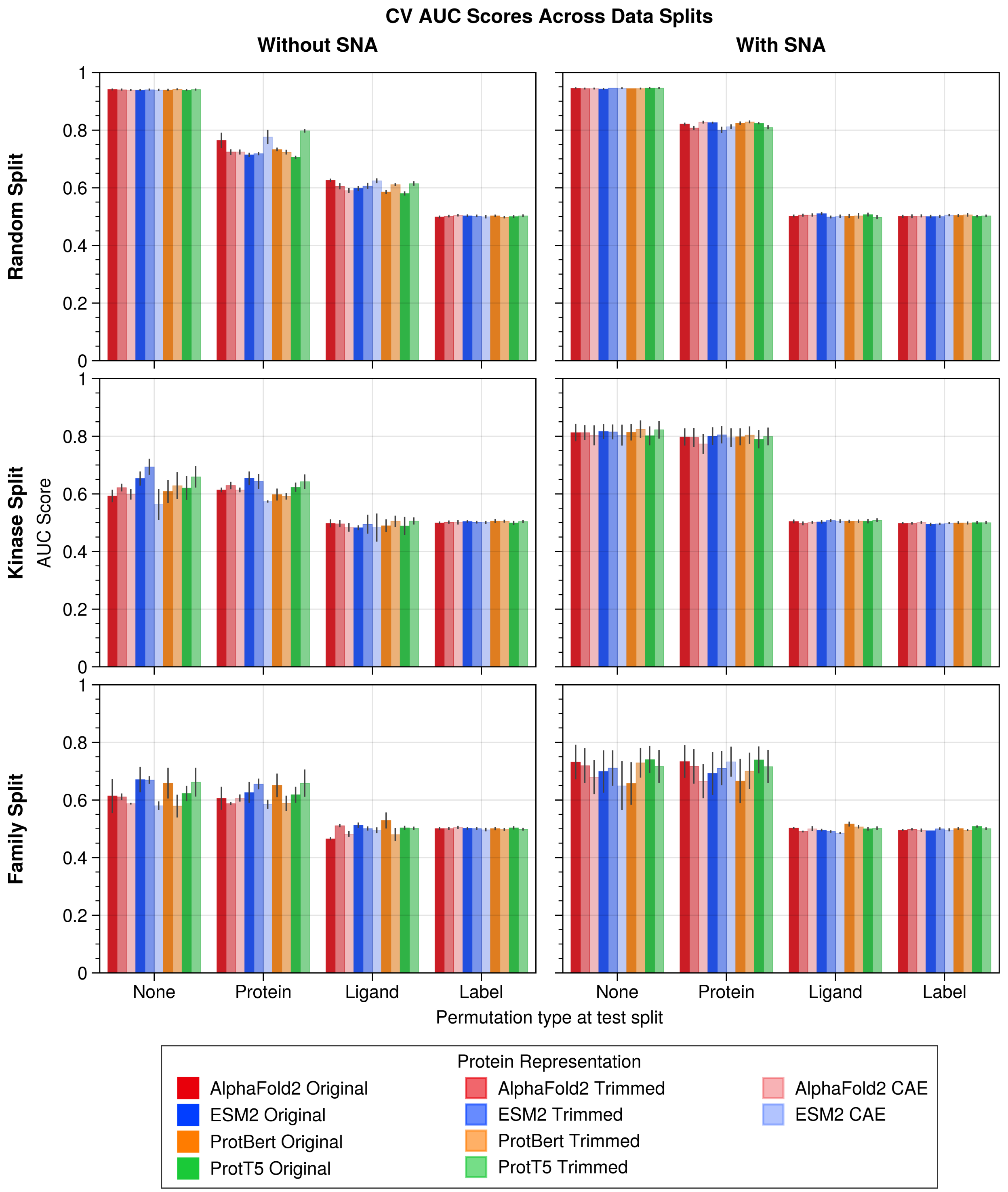

Figure S10: Performance of XGBoost models trained to predict bioactivity of kinase-ligand pairs as measured by AUROC (mean ± s.e.) across cross-validation folds with permutations on the test set and compared with the baseline (no permutation; "None"). Different cross-validation splits were created using either random split (top row), kinase split (middle row) or family split (bottom row) strategies. Models were trained with the dataset as is (left column) or after rebalancing with SNA (right column) using a ratio of 1:1 inactives to actives.

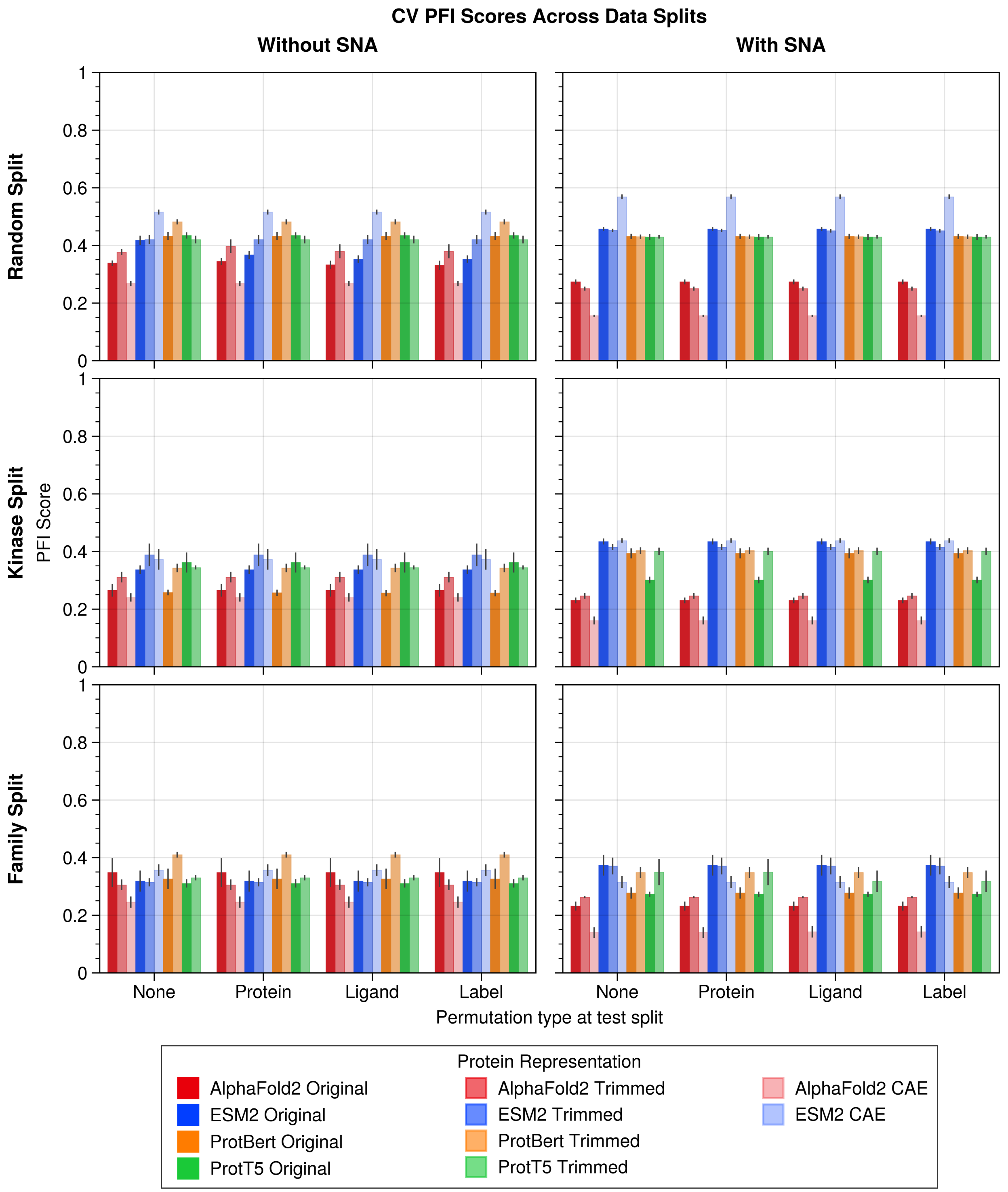

Figure S11: Performance of XGBoost models trained to predict bioactivity of kinase-ligand pairs as measured by PFI (mean ± s.e.) across cross-validation folds with permutations on the test set and compared with the baseline (no permutation; "None"). Different cross-validation splits were created using either random split (top row), kinase split (middle row) or family split (bottom row) strategies. Models were trained with the dataset as is (left column) or after rebalancing with SNA (right column) using a ratio of 1:1 inactives to actives.

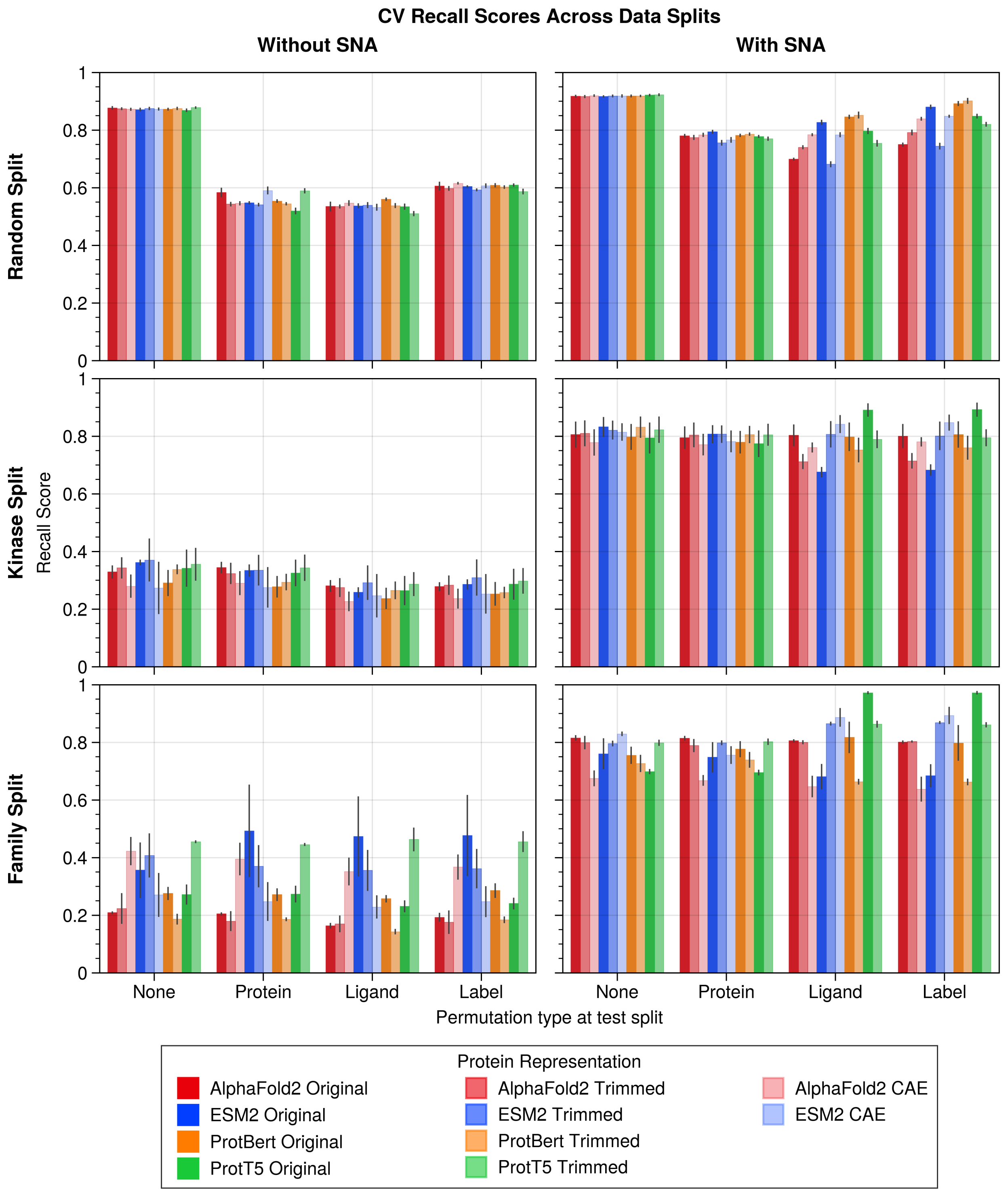

Figure S12: Performance of XGBoost models trained to predict bioactivity of kinase-ligand pairs as measured by Recall (mean ± s.e.) across cross-validation folds with permutations on the test set and compared with the baseline (no permutation; "None"). Different cross-validation splits were created using either random split (top row), kinase split (middle row) or family split (bottom row) strategies. Models were trained with the dataset as is (left column) or after rebalancing with SNA (right column) using a ratio of 1:1 inactives to actives.

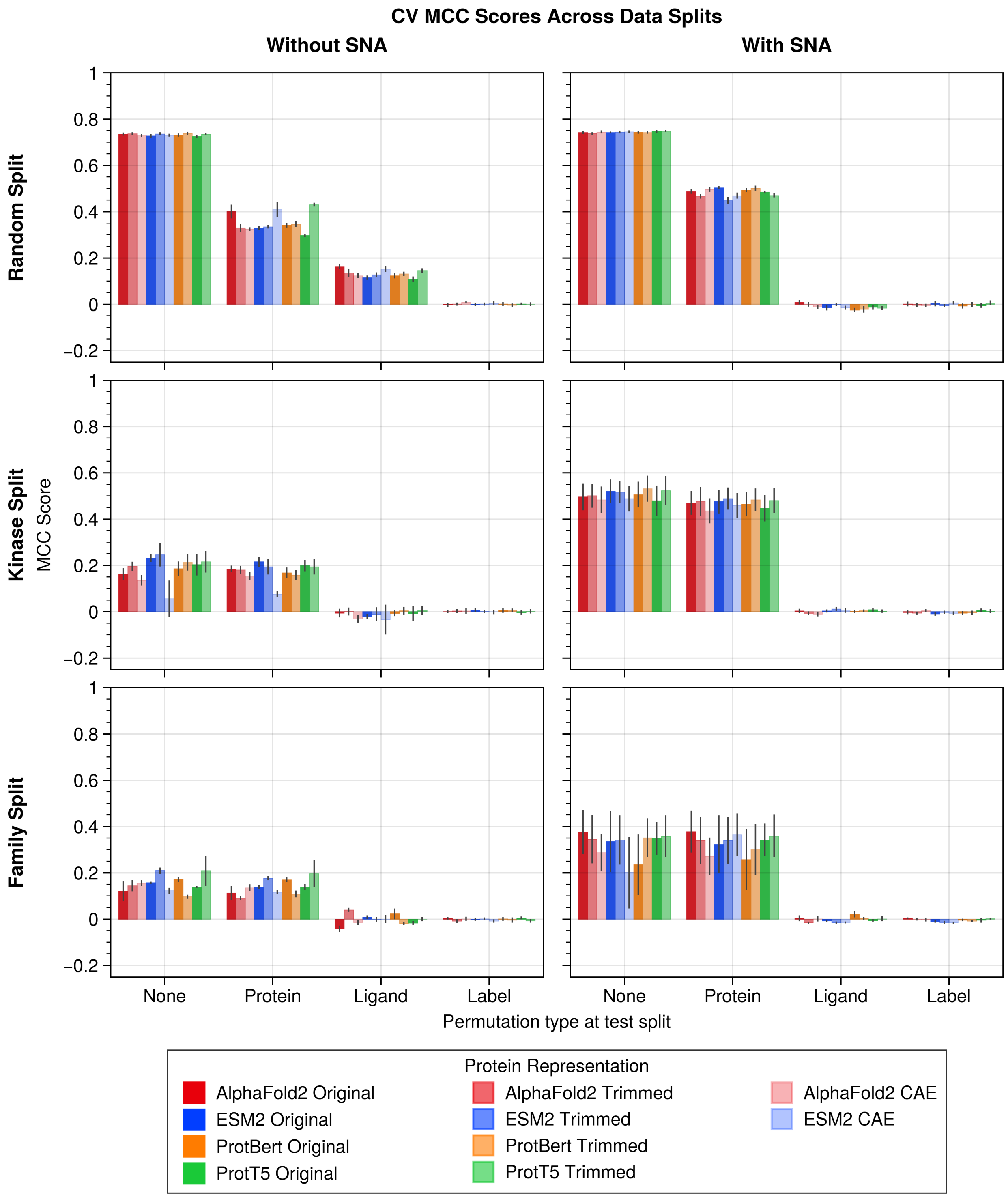

Figure S13: Performance of XGBoost models trained to predict bioactivity of kinase-ligand pairs as measured by MCC (mean ± s.e.) across cross-validation folds with permutations on the test set and compared with the baseline (no permutation; "None"). Different cross-validation splits were created using either random split (top row), kinase split (middle row) or family split (bottom row) strategies. Models were trained with the dataset as is (left column) or after rebalancing with SNA (right column) using a ratio of 1:1 inactives to actives.

References

(S1) Liu, T.; Lin, Y.; Wen, X.; Jorissen, R. N.; Gilson, M. K. BindingDB: a web-accessible database of experimentally determined protein-ligand binding affinities. *Nucleic Acids Research* **2007**, *35*, D198–D201, DOI: 10.1093/nar/gkl999.

(S2) Gaulton, A.; Bellis, L. J.; Bento, A. P.; Chambers, J.; Davies, M.; Hersey, A.; Light, Y.; McGlinchey, S.; Michalovich, D.; Al-Lazikani, B.; Overington, J. P. ChEMBL: a large-scale bioactivity database for drug discovery. *Nucleic Acids Research* **2012**, *40*, D1100–D1107, DOI: 10.1093/nar/gkr777.

(S3) Koleti, A.; Terryn, R.; Stathias, V.; Chung, C.; Cooper, D. J.; Turner, J. P.; Vidovi, D.; Forlin, M.; Kelley, T. T.; DUrso, A.; Allen, B. K.; Torre, D.; Jagodnik, K. M.; Wang, L.; Jenkins, S. L.; Mader, C.; Niu, W.; Fazel, M.; Mahi, N.; Pilarczyk, M.; Clark, N.; Shamsaei, B.; Meller, J.; Vasiliauskas, J.; Reichard, J.; Medvedovic, M.; Maayan, A.; Pillai, A.; Schürer, S. C. Data Portal for the Library of Integrated Network-based Cellular Signatures (LINCS) program: integrated access to diverse large-scale cellular perturbation response data. *Nucleic Acids Research* **2018**, *46*, D558–D566, DOI: 10.1093/nar/gkx1063.

(S4) Christmann-Franck, S.; Van Westen, G. J. P.; Papadatos, G.; Beltran Escudie, F.; Roberts, A.; Overington, J. P.; Domine, D. Unprecedently Large-Scale Kinase Inhibitor Set Enabling the Accurate Prediction of CompoundKinase Activities: A Way toward Selective Promiscuity by Design? *Journal of Chemical Information and Modeling* **2016**, *56*, 1654–1675, DOI: 10.1021/acs.jcim.6b00122, Number: 9.

(S5) Duong-Ly, K. C.; Devarajan, K.; Liang, S.; Horiuchi, K. Y.; Wang, Y.; Ma, H.; Peterson, J. R. Kinase Inhibitor Profiling Reveals Unexpected Opportunities to Inhibit Disease-Associated Mutant Kinases. *Cell Reports* **2016**, *14*, 772–781, DOI: 10.1016/j.celrep.2015.12.080.

(S6) Kalliokoski, T.; Kramer, C.; Vulpetti, A.; Gedeck, P. Comparability of Mixed IC50 Data A Statistical Analysis. *PLoS ONE* **2013**, *8*, e61007, DOI: 10.1371/journal.pone.0061007.

(S7) Akiba, T.; Sano, S.; Yanase, T.; Ohta, T.; Koyama, M. Optuna: A Next-generation Hyperparameter Optimization Framework. Proceedings of the 25th ACM SIGKDD International Conference on Knowledge Discovery & Data Mining. Anchorage AK USA, 2019; pp 2623–2631, DOI: 10.1145/3292500.3330701.
